# Mode and dynamics of *vanA*-type vancomycin resistance dissemination in Dutch hospitals

**DOI:** 10.1186/s13073-020-00825-3

**Published:** 2021-01-20

**Authors:** Sergio Arredondo-Alonso, Janetta Top, Jukka Corander, Rob J. L. Willems, Anita C. Schürch

**Affiliations:** 1 Department of Medical Microbiology, University Medical Center Utrecht, Utrecht, The Netherlands; 2 Department of Biostatistics, University of Oslo, Oslo, Norway; 3 Pathogen Genomics, Wellcome Trust Sanger Institute, Cambridge, CB10 1SA UK; 4 Department of Mathematics and Statistics, Helsinki Institute of Information Technology (HIIT), FI-00014 University of Helsinki, Helsinki, Finland

**Keywords:** *Enterococcus faecium*, Genome sequencing, Vancomycin resistance, Network, Clonal dissemination, Horizontal dissemination, Horizontal gene transfer

## Abstract

**Background:**

*Enterococcus faecium* is a commensal of the gastrointestinal tract of animals and humans but also a causative agent of hospital-acquired infections. Resistance against glycopeptides and to vancomycin has motivated the inclusion of *E. faecium* in the WHO global priority list. Vancomycin resistance can be conferred by the *vanA* gene cluster on the transposon Tn*1546*, which is frequently present in plasmids. The *vanA* gene cluster can be disseminated clonally but also horizontally either by plasmid dissemination or by Tn*1546* transposition between different genomic locations.

**Methods:**

We performed a retrospective study of the genomic epidemiology of 309 vancomycin-resistant *E. faecium* (VRE) isolates across 32 Dutch hospitals (2012–2015). Genomic information regarding clonality and Tn*1546* characterization was extracted using hierBAPS sequence clusters (SC) and TETyper, respectively. Plasmids were predicted using gplas in combination with a network approach based on shared k-mer content. Next, we conducted a pairwise comparison between isolates sharing a potential epidemiological link to elucidate whether clonal, plasmid, or Tn*1546* spread accounted for *vanA*-type resistance dissemination.

**Results:**

On average, we estimated that 59% of VRE cases with a potential epidemiological link were unrelated which was defined as VRE pairs with a distinct Tn*1546* variant. Clonal dissemination accounted for 32% cases in which the same SC and Tn*1546* variants were identified. Horizontal plasmid dissemination accounted for 7% of VRE cases, in which we observed VRE pairs belonging to a distinct SC but carrying an identical plasmid and Tn*1546* variant. In 2% of cases, we observed the same Tn*1546* variant in distinct SC and plasmid types which could be explained by mixed and consecutive events of clonal and plasmid dissemination.

**Conclusions:**

In related VRE cases, the dissemination of the *vanA* gene cluster in Dutch hospitals between 2012 and 2015 was dominated by clonal spread. However, we also identified outbreak settings with high frequencies of plasmid dissemination in which the spread of resistance was mainly driven by horizontal gene transfer (HGT). This study demonstrates the feasibility of distinguishing between modes of dissemination with short-read data and provides a novel assessment to estimate the relative contribution of nested genomic elements in the dissemination of *vanA-*type resistance.

**Supplementary Information:**

The online version contains supplementary material available at 10.1186/s13073-020-00825-3.

## Background

*Enterococcus faecium* is commonly inhabiting the gut of animals and humans but has also emerged as a nosocomial pathogen causing a sizable fraction of healthcare-associated infections, specifically device-associated infections like central line-associated bloodstream and surgical site infections [1, 2]. The intrinsic and acquired multi-drug resistance against fluoroquinolones, aminoglycosides, and more importantly against glycopeptides motivated the inclusion of *E. faecium* in the WHO global priority list [3]. The number of strains resistant against vancomycin, a first-line glycopeptide antibiotic to treat infections with multi-drug-resistant Gram-positive pathogens, dramatically increased first in the USA in the 1990s, followed by other parts of the world [4]. Resistance against vancomycin can be acquired through eight different gene clusters (*vanA*, *vanB*, *vanD*, *vanE*, *vanG*, *vanL*, *vanM*, and *vanN*) [5, 6] of which *vanA* and *vanB*, associated to transposon sequences Tn*1546* and Tn*1549*, respectively, are the predominant vancomycin resistance gene clusters [7].

Clonal spread of vancomycin-resistant *Enterococcus faecium* (VRE) has been extensively described using a plethora of molecular typing schemes. They range from fingerprint-based methods like pulsed-field gel electrophoresis [8] to PCR-based methods such as multiple-locus variable number tandem repeat analysis [9], multilocus sequence typing [10], and whole-genome sequencing [11]. However, due to the fact that vancomycin resistance genes are located on mobile genetic elements, vancomycin resistance also has the potential to be transferred horizontally. In fact, mobilization of the *vanA* gene cluster via insertion in different plasmid backbones has already been reported [12, 13]. To identify the dissemination of *vanA* plasmids within hospital settings, whole-genome sequencing (WGS) based on short-read technologies has been recently applied to collections of hundred hospitalized patient isolates in Denmark and Australia [14, 15]. These studies undertook a reference-based approach to map short-reads against complete plasmids from a selection of isolates. However, this approach can overestimate the presence of a reference plasmid by neglecting the mosaicism observed in these types of sequences as previously observed for *Enterobacteriaceae* isolates [16] and *Enterococcus* populations [17].

The dissemination of the *vanA* gene cluster can occur vertically, in which case the same plasmid type and Tn*1546* variant are observed in two clonal isolates. However, the *vanA* gene cluster can also be horizontally transferred, by two different processes: (i) plasmid dissemination which is reflected by observing the same plasmid type and Tn*1546* variant in strains that have a different clonal background and (ii) transposition of Tn*1546* between different plasmid types [18–20]. This nested nature of these mobile genomic elements resembles the Russian-Doll model which has been previously used to describe the transfer of carbapanamese genes, *bla*_kpc_, in *Enterobacteriaceae* [16]. It is important to note that the Tn*1546* is a non-conjugative transposon but its mobilization can occur when the element is embedded in another conjugative element. Furthermore, the presence of IS elements (e.g., IS*1216*) surrounding the transposon can mobilize the *vanA* gene cluster to other genomic locations [18–20].

The genomic approach conducted here allowed us to fully reconstruct and quantify the most likely mode of dissemination by characterizing the clonal background (hierBAPS SC), *vanA* plasmid type (de novo prediction and network assignment), and Tn*1546* variants harboring the *vanA* resistance gene cluster. Clonal dissemination, defined by vertical inheritance of the same SC, *vanA* plasmid type, and Tn*1546* variant, was the most frequent scenario of vancomycin resistance dissemination occurring in the Netherlands between 2012 and 2015. However, we also detected outbreak settings in which Tn*1546* transposition between distinct plasmid types and/or plasmid dissemination were the dominant mechanisms driving the *vanA* gene cluster dissemination. To our knowledge, we provide one of the first studies to estimate the frequencies of clonal and HGT processes in the dissemination of the *vanA* gene cluster occurring in Dutch healthcare settings between 2012 and 2015.

## Methods

### Dutch VRE collection, short-read WGS, and genome assembly

The isolates from this collection represent a subset of isolates from a previous study we conducted and that consisted of 1644 *E. faecium* isolates [21]. Isolates with the *vanA*-type vancomycin resistance gene cluster (*n* = 309) from 32 Dutch hospitals collected between 2012 and 2015 were further analyzed. DNA extraction and whole-genome sequencing using Illumina NextSeq were conducted as previously described [22]. Short-reads were trimmed using Trim Galore (version 0.6.4_dev) using the flag “--paired” and specifying a phred score of 20 with the flag “--quality” [23]. We used Unicycler (version 0.4.7) passing the short paired-end trimmed reads from Trim Galore [23], a wrapper script that combines cutadapt and fastqc [24, 25]. Unicycler was run specifying the normal mode (--mode) [26]. Unicycler was used to compute the assembly graph provided in the file “assembly.gfa” which selects for the k-mer size that optimizes the ratio between number of dead-ends and contig size in the graph given by SPAdes (version 3.14.0) [27]. For six isolates (E7931, E7942, E8155, E8387, E8437, E9000), Unicycler failed to generate a short-read assembly. In silico prediction using Abricate (https://github.com/tseemann/abricate, version 0.8), with the ResFinder database (indexed on 16th of July 2018) [28] was conducted to search and select for isolates bearing the *vanA* resistance gene.

### Population structure of VREfm isolates

Recombination events and estimation of sequence clusters, in the 309 VRE isolates, were performed using BratNextGen and hierBAPS and retrieved from Arredondo-Alonso et al. [21]. PopPUNK (version 2.0.1) was run specifying the flag “--easy-run” with a minimum k-mer size of 13 (flag --min-k) and creating the files required to generate a microreact project (flag --microreact) [29].

### De novo plasmid prediction

Gplas (version 0.6.1) was used to de novo predict the plasmids present in the assembly graph of each VRE isolate [30]. Gplas was run using mlplasmids [22] as a classifier to predict plasmid sequences (flag “-c”), specifying the species model “*Enterococcus faecium*” (flag -s), a modularity threshold of 0.1 to partition the resulting bins (flag -q), and 50 walks per plasmid seed (flag -x).

### Tn*1546* characterization

TETtyper (unique version) was used [31] to detect SNPs and deletions present in the Tn*1546* sequences of each complete plasmid sequence or predicted *vanA* plasmid bin against a reference sequence (--ref) corresponding to the original transposon structure (NCBI Nucleotide Accession M97297) described by Arthur et al. [32]. We passed the trimmed reads to TETyper with otherwise default parameters.

### Network of complete plasmid sequences and plasmid type definition

Mash (version 2.2.2) [33] specifying a k-mer size (-k) of 21 and sketch size (-s) of 1000 was used to perform k-mer pairwise comparisons between complete plasmid sequences carrying the *vanA* gene cluster. For this purpose, we included the 26 complete vancomycin-resistant (*vanA*) plasmids from the same collection of 1644 *E. faecium* isolates [21] and 60 complete plasmid sequences carrying the Tn*1546* (NCBI Nucleotide Accession M97297) from the PLSDB database [34]. Sequences were retrieved from the PLSDB database with the function Mash screen (max. *p* value = 0.1, Min. Identity = 0.99). We removed PLSDB sequences already present in our set of 26 complete vancomycin-resistant plasmids.

Based on the density distribution of Mash distances, we estimated an optimal cutoff of 0.025 to define the minimum distance to draw an edge between two nodes (complete plasmid sequences) in a network. The igraph R package (version 1.2.4) was considered to represent the network [35]. Independent components (subgraphs, size > 1 node) in the network were considered as plasmid types (A–H). Singleton sequences in the network (NC_014726.1, AP022823.1, NC_013317.1, NZ_CP012594.1, NC_005054.1, NZ_CP022486.1, NZ_CP014531.1, NZ_CP040238.1, E8172_3, NZ_CP036247.1, NZ_CP018130.1, NZ_CP019973.1) were not considered to define plasmid types but were included in all subsequent analyses.

To perform average nucleotide identity (ANI) measures between the complete plasmid sequences, we used the script “average_nucleotide_identity.py” provided in the pyani tool (version 0.2.10) [36] with default parameters. We further considered the reported ANIm alignment coverage and ANIm identity values to support plasmid type assignments. We used the ward.D2 method implemented in the function hclust of the stats R package (version 3.5.1) to cluster the ANIm alignment coverage. Next, we used the function heatmap.2 from the gplots R package (version 3.0.1.1) to integrate the plasmid types previously defined using our proposed network approach with the clustering based on the ANIm alignment coverage.

For visualization purposes, the starting coordinate position of complete *vanA* plasmids was adjusted using the function fixstart from circlator (version 1.5.5) using a customized database of known plasmid replication initiator sequences [37]. Easyfig (version 2.2.2) [38] with a minimum 80% identity and minimum block length of 500 bp were considered to visualize the blastn alignment [39] produced using the complete plasmid sequences belonging to the plasmid type B.

### Network of predicted plasmid bins and integration of plasmid types

The predicted plasmid bins reported by gplas and bearing the *vanA* gene cluster were pairwise compared using Mash (*k* = 21, *s* = 1000). The igraph R package (version 1.2.4) was used to represent a network in which nodes corresponded to *vanA* plasmid bins and edges to connections between bins with a Mash distance lower than 0.025. The same threshold (0.025) to define an edge was considered since the density distribution of Mash distances followed the same pattern as previously observed with the complete plasmid sequences. Furthermore, we computed the total number of components and the average component size using different thresholds to estimate an optimal edge cutoff. The network consisted of 270 nodes and 16 independent components (subgraphs with > 1 node). Component 3 (144 nodes) was partitioned into 3 different graph groups based on its modularity value (0.42) using the function “cluster_louvain” from the igraph R package (version 1.2.4). We focused on components/graph bins with > 10 isolates that were termed as plasmid bin groups (1–8).

Next, we integrated the plasmid types (A–I) and the singleton sequences (NC_014726.1, AP022823.1, NC_013317.1, NZ_CP012594.1, NC_005054.1, NZ_CP022486.1, NZ_CP014531.1, NZ_CP040238.1, E8172_3, NZ_CP036247.1, NZ_CP018130.1, NZ_CP019973.1) into the network of predicted plasmid bins. Then, we computed Mash distances (*k* = 21, *s* = 1000) between complete plasmid sequences and predicted plasmid bins. The igraph R package (version 1.2.4) was used to represent a network in which nodes either corresponded to *vanA* plasmid bins or complete plasmid sequences and edges to connections between sequences (bins or complete sequences) with a Mash distance lower than 0.025. The network consisted of 348 nodes: 270 predicted plasmid bins and 78 complete plasmid sequences. Plasmid bin groups with edges connecting to complete plasmid sequences were assumed to carry the same plasmid type (A, B, C, D, E, I). Only the plasmid bin group J presented no edges connecting to complete plasmid sequences and it was considered as carrying a novel plasmid type.

### Contribution of nested genomic elements in the dissemination of *vanA*-type gene cluster

Pairwise comparisons (e.g., 10 isolates, 45 unique pairs) were computed between VRE isolates sampled within 12 consecutive months and isolated: (i) country-wide, (ii) at the same Dutch region, and (iii) at the same hospital. To avoid grouping the isolates based exclusively on their isolation year, we computed all the windows of 12 months between two consecutive years (e.g., January 2012–January 2013, …, December 2012–December 2013). Only windows with more than 10 isolates were considered to estimate the frequency of *vanA* dissemination. For each remaining window, we determined which genomic elements were shared between pairs of VRE isolates and defined the following scenarios: (i) clonal dissemination, characterized by identical SC, *vanA* plasmid type, and Tn*1546* structure; (ii) HGT plasmid dissemination, characterized by identical *vanA* plasmid type and Tn*1546* structure but distinct SC type; (iii) clonal spread mediated by Tn*1546* mobilization between plasmid types co-existing in the same SC, characterized by identical SC and Tn*1546* structure but distinct plasmid type; (iv) Tn*1546* mobilization between distinct SC and plasmid types, characterized by distinct SC and plasmid types but identical Tn*1546* structure; and (v) no linkage (unrelated cases), distinct Tn*1546* structure. For every two consecutive years (2012–2013, 2013–2014, 2014–2015), we averaged the frequency of the observed scenarios in each of the windows (> 10 isolates).

### Visualization of genomic elements

GADM (version 3.6) [39] was used to download the map with regional borders of the Netherlands and was combined with the R packages ggplot2 (version 3.1.0) and scatterpie (version 0.1.5) (https://github.com/GuangchuangYu/scatterpie) to plot spatial information and SC distribution of the isolates. The R package genoplotR (version 0.8.9) [40] was used to visualize the gene structure and to highlight the *vanA* gene cluster location and the presence of IS elements present in the plasmid types (A–F). The R package ggtree (version 1.14.6) [41] was used to integrate the neighbor-joining tree based on the core genome given by PopPUNK together with SC assignment and predicted *vanA* plasmid types. In addition, a Microreact project (version 15.0.0) [42] was created to integrate and visualize the genomic and metadata information.

An RMarkdown document is provided to integrate all the code and reproduce the results generated for all the methods section described above [43].

## Results

### The population structure of VRE from Dutch hospitals

This study was conducted with samples from an extensive collection of 1644 *E. faecium* isolates derived from clinical and non-clinical sources with associated short-read WGS data [21]. We focused on Dutch clinical isolates with complete metadata information regarding clinical settings and isolation date, from 2012 to 2015 (*n* = 593). From this selection, 309 (52.1%) and 265 (44.7%) isolates carried the *vanA* and/or *vanB* gene clusters, respectively. We focused on the *vanA* VRE samples since the resistance gene cluster is frequently present on plasmids [44, 45]. This permitted us to investigate a nested genomic system in which the glycopeptide resistance can be disseminated on a clonal, plasmid, and/or transposon level. These 309 VRE samples were isolated from patients in 32 Dutch hospitals.

**Fig. 1:**
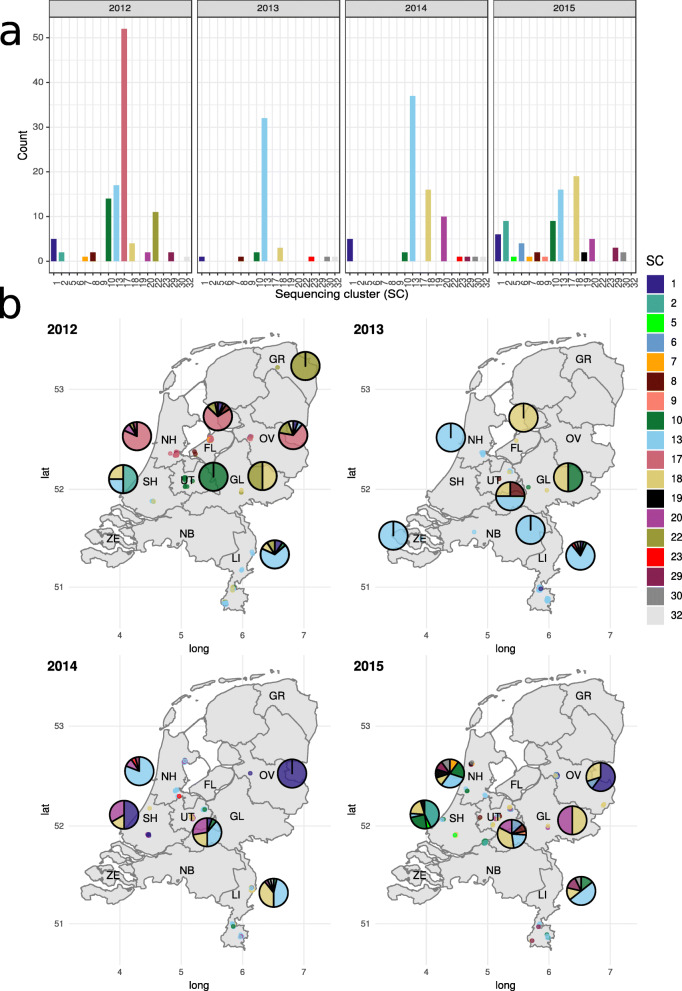
Temporal and spatial distribution of the 309 VRE isolates per isolation year (2012–2015). Isolates were colored according to their associated hierBAPS sequencing clusters (SC) (*n* = 18). **a** Count of the total number of isolates grouped in each hierBAPS sequencing cluster (SC). **b** For each isolate, the latitude and longitude of the hospital where the sample was processed are indicated. To mitigate overlapping, the points representing the isolates were jittered and their specific coordinates can be inspected at https://microreact.org/project/FCUD_d1zt. For each region, a pie chart showing the distribution of SCs is indicated to facilitate the inspection of the results. The names of the Dutch regions with isolates present are abbreviated in the following manner: FL Flevoland, GL Gelderland, GR Groningen, LI Limburg, NB North-Brabant, NH North-Holland, OV Overijssel, SH South-Holland, UT Utrecht, ZE Zeeland

The clonality of these 309 *vanA* VRE samples was determined using hierBAPS [46], after filtering for recombination events as previously described [21]. HierBAPS defined 18 different sequence clusters (SCs) of which SC13 (*n* = 102, 33%), SC17 (*n* = 52, 16.8%), and SC18 (*n* = 42, 13.6%) represented the most predominant clones in the dataset (Fig. 1a). The distribution of these SC across time and geographical position showed that SC13 was widespread in Dutch hospitals for the entire collection period (2012–2015) (Fig. 1b) compared to SC17 which was observed in distinct regions (North-Holland, Flevoland, Overijssel) from August–September 2012 (Fig. 1b). SC18 was detected around 2014 and 2015 in several Dutch regions (Fig. 1b). We computed a core-genome-based neighbor-joining tree of the samples using PopPUNK [29] for 303 isolates (98.1%) with an associated short-read assembly [29], and the tree was combined with metadata information in the following Microreact project https://microreact.org/project/FCUD_d1zt. Metadata information and SC assignment of all VRE isolates (*n* = 309) are also available in Additional File 1: Table S1.

### Developing a novel plasmid typing scheme based on network clustering

To establish a partitioning scheme similar to hierBAPS SCs but uniquely based on the similarity between plasmids carrying the *vanA*-type gene cluster, we first retrieved 26 *E. faecium* complete *vanA* plasmids from the same collection of 1644 clinical and non-clinical isolates [21] with known isolation date and country (Table 1). These 26 *vanA* complete plasmids were previously completed using short- and long-read sequencing data and were previously published in Arredondo-Alonso et al. [21]. To obtain a global picture of the diversity of *vanA* plasmids, we included 60 PLSDB plasmid sequences carrying the Tn*1546* sequence.

**Table 1:** Metadata information of the isolates carrying the *vanA* complete plasmid sequences (*n* = 25) described in Arredondo-Alonso et al. [21] and used to define the plasmid types: A, B, C, D, E, F

Plasmid ID	Plasmid type	Country^a^	Isolation Year	Isolation source	SC type^b^	Between coverage^c^ (%)	Within coverage^d^ (%)
E0139_2	A	the Netherlands	1996	Non-hospital	29	38	85
E0595_2	A	the Netherlands	1996	Pig	30		
E0656_2	A	the Netherlands	1999	Non-hospital	30		
E6975_4	B	Greece	2010	Hospital	13	25	76
E7114_4	B	Latvia	2010	Hospital	13		
E7160_5	B	Slovenia	2009	Hospital	5		
E7240_4	B	Greece	2010	Hospital	10		
E7246_6	B	Greece	2009	Hospital	13		
E7471_5	B	the Netherlands	2012	Hospital	22		
E8423_3	B	the Netherlands	2015	Hospital	18		
E7313_3	C	the Netherlands	2012	Hospital	2	37	80
E8014_3	C	the Netherlands	2014	Hospital	13		
E8414_4	C	the Netherlands	2014	Hospital	1		
E6055_4	D	Portugal	2010	Hospital	22	34	73
E8040_4	D	the Netherlands	2014	Hospital	1		
E8202_3	D	the Netherlands	2015	Hospital	1		
E4227_3	E	Sweden	2005	Chicken	35	29	68
E4239_3	E	Sweden	2007	Chicken	35		
E6020_3	F	Latvia	2010	Hospital	8	35	67
E6988_5	F	Latvia	2010	Hospital	21		
E7025_5	F	Latvia	2010	Hospital	23		
E7040_5	F	Latvia	2010	Hospital	21		
E7067_5	F	Latvia	2010	Hospital	21		
E7070_9	F	Latvia	2010	Hospital	21		
E7207_6	F	Greece	2008	Hospital	3		

^a^Country of sampling isolation

^b^hierBAPS SC. Clonal background of the isolate carrying the plasmid sequence

^c^Between coverage refers to the average coverage resulting from pairwise comparisons of complete plasmid sequences belonging to different plasmid types (A–F)

^d^Within coverage refers to the average coverage resulting from pairwise comparisons of complete plasmid sequences belonging to the same plasmid type

**Fig. 2:**
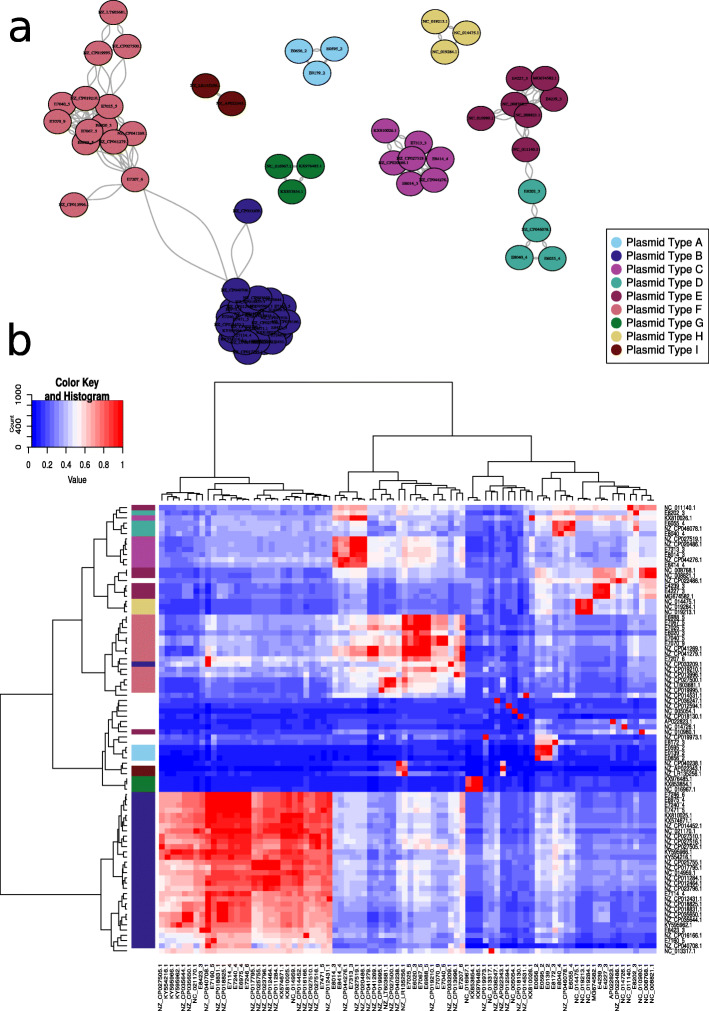
Definition of the plasmid types (A–I) observed in the 86 retrieved *vanA* complete plasmid sequences. **a** Network representation of the *vanA* complete plasmid sequences based on Mash distances (*k* = 21, *s* = 1000). Nodes (*n* = 76) in the network correspond to complete plasmid sequences and edges were defined if nodes shared a minimum Mash distance of 0.025. Edges correspond to connections between sequences with a similar k-mer composition. Nodes were colored according to the defined plasmid type assignment. Based on the modularity values and structure of the network components, we identified the plasmid types: A, B, C, D, E, G, H, and I. Twelve complete plasmid sequences (NC_014726.1, AP022823.1, NC_013317.1, NZ_CP012594.1, NC_005054.1, NZ_CP022486.1, NZ_CP014531.1, NZ_CP040238.1, E8172_3, NZ_CP036247.1, NZ_CP018130.1, NZ_CP019973.1) are not present in the network since they did not share any edges connecting to other nodes (singletons). **b** Heatmap and hierarchical clustering (ward.D2 method) of the pyani pairwise alignment coverage obtained from the 86 *vanA* complete plasmid sequences. On the left side, the plasmid types (A, B, C, D, E, F, G, H, I) previously defined in the network (panel **a**) are indicated. The twelve plasmid sequences corresponding to singletons are also included in this analysis

These complete sequences (*n* = 86) were pairwise compared using Mash (*k* = 21, *s* = 1000) and integrated into a network. Based on the k-mer distance distribution (Additional File 2: Fig. S1), we observed two peaks with different heights and resembling a bimodal distribution. Based on this, an optimal cutoff of 0.025 was used to define an edge in the network. This cutoff also split the minor and major modes (peaks) observed in the k-mer distribution (Additional File 2: Fig. S1). This edge cutoff resulted in seven independent subgraphs in the network. For each of the subgraphs, we split them based on their modularity values to retrieve which nodes (plasmid sequences) were highly interconnected and thus signified distinct *vanA* plasmid types (A–I) with similar content and structure (Fig. 2a). The plasmid types D and E were initially predicted as a single type but were further split based on their average nucleotide coverage (Fig. 2b). Twelve complete plasmid sequences (AP022823.1, E8172_3, NC_005054.1, NC_013317.1, NC_014726.1, NZ_CP012594.1, NZ_CP014531.1, NZ_CP018130.1, NZ_CP019973.1, NZ_CP022486.1, NZ_CP036247.1, NZ_CP040238.1) remained as singletons in the network.

To better understand the modularity and similarity of these plasmid types, we estimated average nucleotide identity (ANI) values using pyani [36]. This allowed retrieving ANI coverage and identity values of the aligned regions between two complete plasmid sequences (pairwise comparisons). We observed that the average coverage between plasmid alignments belonging to the same plasmid type was 79.1% compared to a coverage of 31.6% when comparing alignments from different plasmid types. The hierarchical clustering (ward.D2 method) of the alignment coverage reported by pyani showed a high concordance with the plasmid types inferred in our network approach (Fig. 2b). We observed three particular cases of partial disagreement between the plasmid typing and the coverage clustering. These correspond to the plasmid sequences NC_011140.1 (type E), KX810026.1 (type F), and NZ_CP033209.1 (type B) (Fig. 2b). The position of the nodes representing these sequences in the network approach showed that they were not connected to all members defining the plasmid type. This indicated that these sequences carry k-mer modules not shared in all members of the plasmid type which is also reflected in Fig. 2b.

We did not observe differences in the average identity values between aligned regions within (98.9%) and between (99.7%) plasmid types indicating a common origin of the plasmid modules present in the different types (Additional File 2: Fig. S2). To exemplify this, we show the plasmid modularity observed in the complete sequences from plasmid type B, since it had the highest SC diversity and number of associated sequences (Additional File 2: Fig. S3).

In Additional File 3, we provide a detailed genomic characterization of the plasmid types (A–I) with a focus on (i) replication initiator proteins (rep), (ii) Tn*1546* variant compared to the original sequence described by Arthur et al. [32], (iii) antimicrobial resistance (AMR) genes distinct from the *vanA* gene cluster, and (iv) presence of well-known *E. faecium* plasmid toxin-antitoxin systems such as ω-ε-ζ and axe-txe [47].

### Network of predicted plasmid bins

Next, we performed a de novo plasmid prediction of the sequences carrying the *vanA* gene cluster in the set of 303 VRE samples with an associated short-read assembly using gplas [30]. This tool uses a combination of machine-learning and a graph-based approach to predict plasmid sequences from short-read graphs [30]. Contigs predicted as belonging to the same plasmid sequence are returned in the same bin.

In 282 isolates (93.1%), the contig which encodes for the *vanA* gene cluster (*vanA* contig) was present in a plasmid bin predicted by gplas. In the remaining isolates (*n* = 21, 6.9%), the *vanA* contig remained unbinned and thus we could not predict whether the *vanA* gene cluster was part of a plasmid. Unbinning of the *vanA* contig could be caused by a fragmented assembly graph due to, for example, a low sequence coverage. Also, high differences in the k-mer coverage of contigs from the same plasmid can prohibit binning of plasmid contigs with gplas. The inability of gplas to predict the plasmid location of *vanA* could also be indicative of a chromosomal location of the *vanA* gene cluster. However, manual inspection of the assembly graph in these 21 isolates revealed that the *vanA* k-mer coverage was clearly higher and distinct from the median k-mer coverage of all contigs, indicative of a plasmid location with a higher copy number compared to the chromosome.

Based on these findings, we concluded that in all 303 *vanA* VRE isolates with a short-read assembly (100%), the gene cluster was present in a plasmid background. The preferential presence of the *vanA* cluster in a plasmid was previously reported by Freitas et al. (53 isolates, 100% plasmid encoded *vanA*) [44] and Wardal et al. (88 isolates, 98% plasmid encoded *vanA*) [45]. These 27 isolates in which gplas could not assign the *vanA* gene cluster to a particular plasmid bin, despite being most likely on a plasmid background were excluded from further analysis.

The 282 isolates containing a predicted gplas plasmid bin were integrated into a network of Mash distances (*k* = 21, *s* = 1000) (Additional File 2: Fig. S4) in which nodes corresponded to predicted plasmid bins and edges to connections of bins with a similar k-mer composition. The distribution of k-mer distances between the plasmid bins (Additional File 2: Fig. S5a) followed the same pattern as observed with the complete plasmid sequences (Additional File 2: Fig. S1). We observed again a bimodal distribution with minor and major modes (Additional File 2: Fig. S5a). The number of resulting components and the average size of the components resulting from different edge thresholds (Additional File 2: Fig. S5b) also indicated that a threshold of 0.025 can be used to draw an edge between nodes in the network shown in Additional File 2: Fig. S4. The network consisted of 270 nodes with 16 different components (> 1 isolate) (Additional File 2: Fig. S4). We observed the presence of a large component (144 nodes) (Additional File 2: Fig. S4, central component) that was split into 3 subgraphs based on its component modularity value (0.42) (Additional File 2: Fig. S4a). In the next analyses, we further focused on components or subgraphs with more than 10 isolates which are indicated in Additional File 2: Fig. S4 and described in Table 2.

**Table 2:** Description of the *vanA* plasmid bin groups (> 10 isolates) present in the plasmid network (270 nodes) consisting uniquely of predicted plasmid bins. In total, eight distinct plasmid bin groups were defined with a total of 239 isolates

*vanA* plasmid bin group^a^	Number of isolates	hierBAPS SC	Plasmid type
1	11	SC10 (100%)	J
2	31	SC13 (93.1%) SC10 (3.4%) SC1 (3.4%)	C
3	76	SC13 (72.0%) SC2 (12.0%) SC1 (4.0%) SC10 (4.0%) SC18 (2.7%) SC9 (1.3%) SC19 (1.3%) SC22 (1.3%) SC23 (1.3%)	C
4	62	SC17 (66.1%) SC10 (17.7%) SC18 (12.9%) SC22 (1.6%) SC23 (1.6%)	B
5	20	SC13 (50.0%) SC17 (40.0%) SC7 (5.0%) SC8 (5.0%)	I
6	17	SC20 (47.1%) SC18 (35.3%) SC1 (11.8%) SC2 (5.9%)	D
7	12	SC29 (41.7%) SC30 (33.3%) SC32 (25.0%)	A
8	10	SC18 (100%)	E

^a^The plasmid bin groups were assigned as carrying a particular plasmid type (A–I) after including the complete *vanA* plasmid sequences into the network and observing co-clustering of predicted and complete plasmid sequences. For each *vanA* plasmid bin group predicted plasmid type, we indicated the total number of isolates belonging to the group forming the predicted plasmid type, percentage of SCs, and assigned plasmid type

### Validating the predicted plasmid network by integration of long-read complete plasmid sequences

**Fig. 3:**
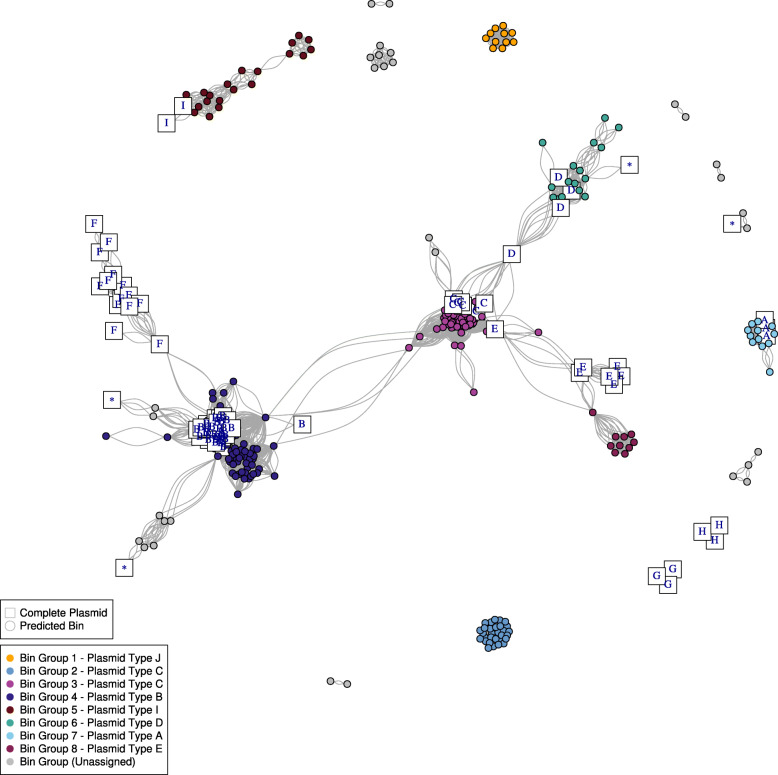
Network representation of the predicted plasmid bins and complete *vanA* plasmid sequences based on Mash distances (*k* = 21, *s* = 1000). The shape of the nodes (*n* = 348) indicates if they correspond to predicted plasmid bins (circle, *n* = 270) or complete plasmid sequences (square, *n* = 78). Nodes corresponding to complete plasmid sequences were named with a single letter based on the assigned s plasmid type (A, B, C, D, E, F, G, H, I) or with an asterisk if they corresponded to sequences defined as singletons in the plasmid typing network (Fig. 2). Edges are connections between nodes sharing a minimum Mash distance of 0.025. Nodes corresponding to predicted plasmid bins (circle) were colored according to their plasmid bin group

To elucidate gene content and synteny of the plasmid bins (*n* = 282), we integrated the complete plasmid sequences (Table 1, Fig. 2) (*n* = 74) used to define the nine plasmid types A to I, and the singleton sequences (*n* = 12) with the predicted plasmid bins carrying the *vanA* gene cluster (*n* = 282). The presence of edges connecting complete plasmids and predicted *vanA* plasmid bins revealed that the predictions had a similar k-mer composition and thus further validated the predicted *vanA* plasmid bins (Additional File 2: Fig. S4). In addition, we included all *E. faecium* public and complete plasmid sequences carrying the *vanA* resistance gene cluster derived from the PLSDB database [34]. This resulted in a network of 348 nodes (270 predicted bins, 78 complete plasmid sequences) and 14 components (> 1 node) (Fig. 3). We observed that the predicted bin groups 3, 4, 6, and 7 were co-clustering together with complete plasmid sequences from the plasmid types C, B, D, and A respectively (Fig. 3). This indicated that these four bin groups had a k-mer composition highly similar to the plasmid types previously defined. In the predicted bin groups 5 and 8, we also observed connections between predicted bin groups and complete plasmid sequences. The predicted bin group 5 had connections to two PLSDB plasmid sequences (NZ_AP022343.1, NZ_LR135256.1) belonging to the plasmid type I (Fig. 3). The predicted bin group 8 had a single node connecting to three PLSDB plasmid sequences from the plasmid type E (NC_008768.1, NC_008821.1, NC_011140.1) (Fig. 3). However, in these two bin groups (5, 8) the number of bins connecting to plasmid sequences was lower than for the previous bin groups (3, 4, 6, 7) which indicated a more heterogeneous composition of the k-mer content for these two plasmid bin groups.

The inclusion of plasmids completed with long-read sequencing data was fundamental to deduce that the plasmid prediction of the isolates from bin group 2 was incorrect. In this bin, one of the isolates (E8014) had an associated complete plasmid sequence (E8014_3, Fig. 2) but we observed no connections between its predicted gplas bin, present at plasmid bin 2, and its associated plasmid sequence (E8014_3, plasmid type C). Based on this, we concluded that the prediction given by gplas was erroneous and the k-mer composition of plasmid bin group 2 should be similar to the plasmid type C despite being disconnected in the network. We only considered the group bin 1 as carrying a novel plasmid type (J) since it was not connected to any complete plasmid sequence (Fig. 3).

This network approach allowed assigning 239 isolates with a particular plasmid type (A, B, C, D, E, I, J) (Table 2). In Additional File 3, we provide an extensive characterization of the plasmid bin groups observed in the network (Fig. 3) based on group diversity regarding SC, Tn*1546* variants, geographical site, and year of isolation.

For the following analyses, we considered all isolates (*n* = 225) for which we had complete metadata information (hospital and isolation date), SC, plasmid type, Tn*1546* assignments, and were present in the PopPUNK core-genome tree.

SC, plasmid type, and Tn*1546* assignments and the nomenclature used to name these transposon variants are outlined in Table 3 and Additional File 4: Table S2.

**Table 3:** Ad hoc scheme used to name the Tn*1546* variants present in the predicted *vanA* plasmid sequences

Type	Position	Nomenclature
SNP	C806T	1
SNP	G3966T	2
SNP	G4351T	3
SNP	T7658C	4
SNP	G7747T	5
SNP	G8234T	6
SNP	C8833T	7
SNP	C9692T	8
Deletion	orf1	M
Deletion	orf2	N
Deletion	*vanR*	R
Deletion	*vanS*	S
Deletion	*vanH*	H
Deletion	*vanA*	A
Deletion	*vanX*	X
Deletion	*vanY*	Y
Deletion	*vanZ*	Z
Deletion	Intergenic	I

**Fig. 4:**
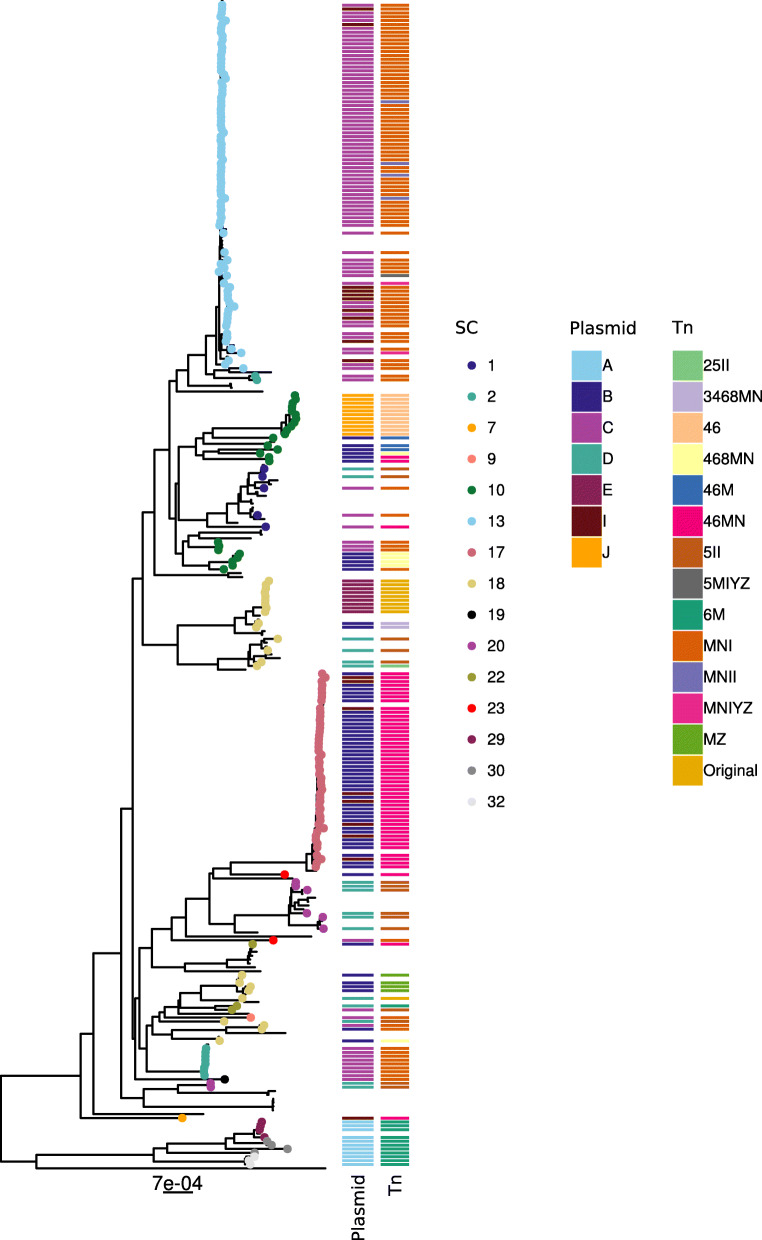
Core-genome-based tree generated by PopPUNK in 303 VRE isolates with an associated short-read assembly. For 225 isolates, we had complete metadata information (hospital and isolation date), hierBAPS SC, plasmid type, and Tn*1546* assignments. The nodes of the core-genome tree corresponding to this subset of 225 isolates are colored based on their associated hierBAPS SC (*n* = 15). The plasmid type (*n* = 6) and Tn*1546* variants (*n* = 14) assignments are plotted in two distinct and adjacent panels. Tn*1546* variants were named following the ad hoc scheme shown in Table 3. This information can also be fully inspected interactively in the following Microreact project: https://microreact.org/project/FCUD_d1zt

In Fig. 4, we combined the core-genome-based neighbor-joining tree with SC, *vanA* plasmid type, and Tn*1546* assignments. Some closely related isolates in the core-genome tree had distinct predicted *vanA* plasmid types as exemplified by SC17 containing plasmid types B and I (Fig. 4). However, these plasmid types contained the same Tn*1546* variant (46MN), which is characterized by deletions of orf1 and orf2 and SNPs T7658C, G8234T. This observation suggested that plasmid types B and I co-resided in the same isolates and that Tn*1546* was internally mobilized between these two plasmid types within the same SC. The isolates belonging to the clonal background SC10 were also closely related in the core-genome tree but shared distinct plasmid types and were associated to different Tn*1546* variants: (i) plasmid type J with 46 variant (SNPs G7747T+C8833T); (ii) plasmid type B associated mostly to 46M, 46MN, and 468MN; (iii) plasmid type C with MNI variant (deletions 1-3417 and 8650-8827). This indicated that isolates from the clonal background SC10 have horizontally acquired different *vanA* plasmids associated with distinct Tn*1546* variants. This situation is also apparent for SC18 isolates which were closely related in the core-genome tree but carried three distinct plasmid types (B, D, E) which were associated with distinct Tn*1546* variants (Fig. 4).

However, we observed overall a high concordance between the SC and Tn*1546* assignments (Fig. 4), which suggested that clonal spread was the main driver of the *vanA* resistance gene cluster dissemination. To facilitate the exploration of the results, SC, plasmid type, and Tn*1546* assignments were also integrated into the Microreact project https://microreact.org/project/FCUD_d1zt

### Dynamics of *vanA*-type resistance dissemination

The identification of the seven *vanA* plasmid types (A, B, C, D, E I, J) present in our Dutch VRE collection allowed us to estimate the contribution of the nested genetic elements, clone (defined as hierBAPS-based SC), plasmid type, and Tn*1546* variant, into the dissemination of the *vanA* gene cluster.

To establish this, we first grouped VRE isolates (*n* = 225) with a potential epidemiological link which corresponded to isolates sampled from two consecutive years. From each year interval, we considered all windows of 12 consecutive months with at least 10 isolates to estimate the dissemination of *vanA* resistance. This approach was undertaken to avoid grouping the isolates based uniquely on their isolation year which neglects the comparison between isolates recovered within a few months. For each window, we performed a pairwise “all vs. all” comparison to define the most likely scenarios of the dissemination of the *vanA* gene cluster (see the section “Contribution of nested genomic elements in the dissemination of *vanA*-type gene cluster” in the “Methods”). We performed the same analysis on Dutch regions (Additional File 2: Fig. S6) and hospitals (Additional File 2: Fig. S7) to identify particular SC, plasmid type, and Tn*1546* variants involved in the dissemination at particular time periods.

**Fig. 5:**
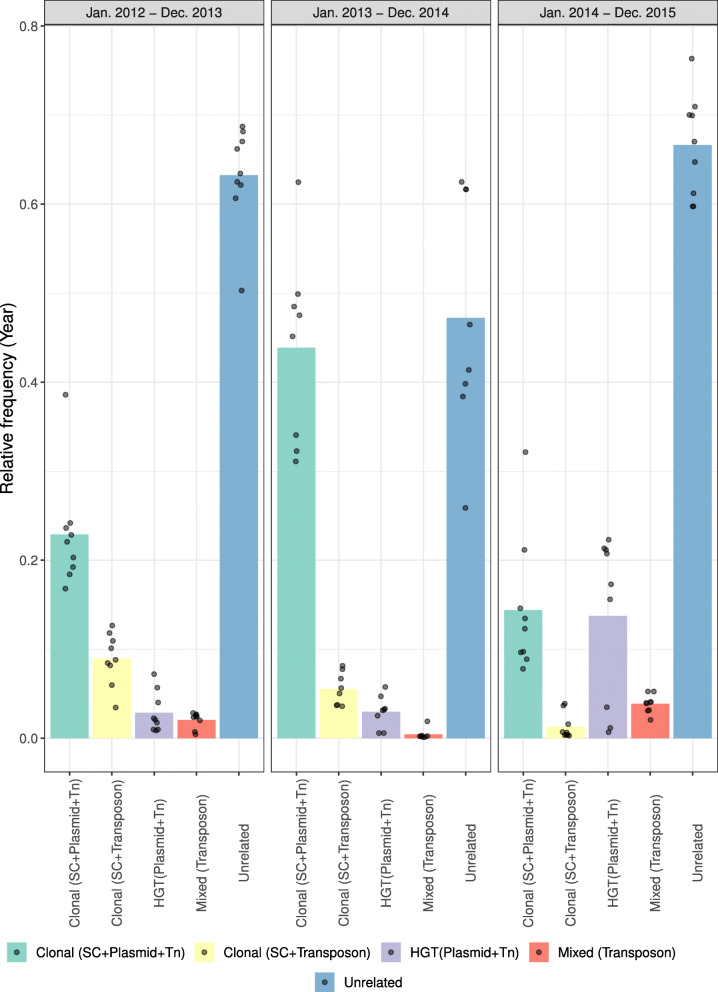
Country-wide contribution of dissemination modes in the spread of *vanA*-type vancomycin resistance in the Netherlands. VRE isolates (*n* = 225) with complete metadata, hierBAPS SC, plasmid type, and Tn*1546* assignments were considered. In this analysis, time intervals of 2 years (January 2012–December 2013; January 2013–December 2014; January 2014–December 2015) were considered to group the isolates. For each time interval, all windows of 12 consecutive months with at least 10 isolates were considered. From each window, we undertook a pairwise comparison to estimate the frequency of the following events: (i) clonal dissemination, pairs of isolates sharing the same hierBAPS SC, *vanA* plasmid type, and Tn*1546* variant; (ii) plasmid dissemination, pairs of isolates sharing the same *vanA* plasmid type and Tn*1546* variant but distinct hierBAPS SC; (iii) clonal dissemination associated with Tn*1546* mobilization, pairs of isolates sharing the same hierBAPS SC and Tn*1546* variant but different *vanA* plasmid type; (iv) Tn*1546* mobilization linked to clonal and horizontal dissemination, pairs of isolates sharing the same Tn*1546* variant and distinct hierBAPS SC and *vanA* plasmid type; and (v) unrelated cases, pairs of isolates with distinct Tn*1546* variants

On a country-wide level, we observed that most of the isolates did not share the same Tn*1546* variant and thus were categorized as unrelated (avg. freq = 0.59) (Fig. 5). Between isolates with an identical Tn*1546* variant, clonal spread defined by observing the same SC, plasmid type, and Tn*1546* was the predominant mode of vancomycin resistance spread (avg. freq = 0.27) and clearly dominated the dissemination of *vanA* between January 2013 and December 2014 (freq = 0.44) (Fig. 5). This clonal spread was accentuated in some regions like Flevoland (January 2012–December 2013) (freq = 0.70) or North-Holland (January 2012–December 2013) (freq = 0.75) in which the clone SC17, associated with plasmid type B and Tn*1546* variant 46MN (deletions in orf1 and orf2 1-3343, SNPs T7658C, G8234T), was responsible for the spread of vancomycin resistance (Additional File 2: Fig. S6). In this last region (North-Holland), the dissemination of the *vanA* cluster was still mainly dominated by clonal spread between January 2013 and December 2014 but was driven by a distinct clone, SC13, associated with the plasmid type C and the Tn*1546* variant MNI (deletions in orf1 and orf2 1-3343 and the intergenic region 8650-8827) (Additional File 2: Fig. S6). We also quantified events of clonal spread (avg. freq = 0.05) (Fig. 5, category “Clonal SC + Transposon”) in which isolates shared identical SC and Tn*1546* variants but carried a distinct plasmid type. These cases most likely correspond to instances of Tn*1546* mobilization between plasmids co-existing in the same isolates as observed in Overijssel (January 2012–December 2013) (freq = 0.32) in which SC17 and the Tn*1546* 46MN variant (deletions in orf1, orf2 1-3343, SNPs T7658C, G8234T) were found mostly in the plasmid types B and I. Overall, clonal spread either by observing the entire nested genomic system or the same SC and Tn*1546* variant had a frequency of ~ 0.32.

Plasmid dissemination characterized by the same plasmid type and Tn*1546* variant but distinct SC (HGT) had an average frequency of 0.07 (Fig. 5). During January 2014 and December 2015, we observed an increase in the frequency of plasmid spread (0.14) and more accentuated in the region of South-Holland (January 2014–December 2015) (Additional File 2: Fig. S6). In this region, we identified four distinct clonal backgrounds (SCs 2, 10, 13, 19) carrying the same plasmid type C with the Tn*1546* MNI variant (deletions in orf1 and orf2 1-3417 and intergenic region 8650-8827).

Finally, we also observed cases of isolates sharing an identical Tn*1546* variant (avg. freq = 0.02) (Fig. 5, category “Mixed Transposon”) on distinct clonal backgrounds and plasmid types. This could be the result of mobilization of Tn*1546* between plasmids from the same SC and posterior plasmid transfer of the *vanA* gene cluster to a distinct SC.

## Discussion

In this study, we propose a graph-based plasmid prediction coupled with a network analysis of the shared plasmid k-mer content to elucidate the dynamics of vertical and horizontal dissemination of AMR genes with short-read WGS. We used this approach to elucidate the mode of *vanA*-type vancomycin resistance dissemination. Clonal and horizontal mobilization of *vanA* were previously documented but the quantity of these events on a large scale is largely unexplored. Our analysis permitted us to distinguish and quantify dissemination occurring by (i) clonal spread of the entire nested system (identical SC, plasmid, Tn*1546*), (ii) plasmid dissemination, (iii) clonal dissemination associated to Tn*1546* mobilization (identical SC, Tn*1546*), and (iv) mixed events of clonal and plasmid dissemination resulting in Tn*1546* mobilization (same transposon variant, distinct SC and plasmid types). This revealed that clonal dissemination was the predominant (~ 32%) mode of spread of *vanA* type of vancomycin followed by plasmid dissemination (~ 7%) and mixed dissemination of Tn*1546* involving different SC and plasmid types (~ 2%). However, we also observed that most of the cases were unrelated (~ 59%) which may indicate distinct and multiple introductions of vancomycin resistance in the Netherlands during 2012 and 2015. The approach presented here can be applied to study clonal and HGT dissemination of other AMR genes, such as carbapanamese genes (*bla*_kpc_) in *Enterobacteriaceae* or colistin resistance genes (*mcr*) in *Escherichia coli*.

Previous studies have described the importance of both clonal and horizontal Tn*1546* dissemination in the emergence of VRE isolates [18, 20]. However, a quantitative assessment of the contribution from the different nested genomic elements in the dissemination of vancomycin resistance has not been previously performed. Combining existing short-read WGS with complete *vanA* plasmids allowed us to define and characterize several plasmid types present in the collection. The integration of previously completed *vanA* plasmids was essential to elucidate the genetic content of the *vanA* plasmid bins present in our predicted network. These *vanA* plasmid types were defined by ~ 99% identity and ~ 79% coverage and were present in different clonal backgrounds (SCs) and carried a predominant Tn*1546* variant that accumulated additional SNPs and/or deletions (Additional File 3). The genomic relatedness of strains, using hierBAPS, plasmid type and Tn*1546* variant calling was combined to sketch a comprehensive picture of the molecular epidemiology of *vanA*-type vancomycin resistance in Dutch hospitals.

Transposition of Tn*1546* between different plasmids has been reported before. Heaton et al. showed the transfer of the Tn*1546* element from a non-conjugative to a conjugative plasmid in the same bacterial cell which was mediated by flanking IS*1216* elements [19]. This event can result in the presence of an identical Tn*1546* variant in distinct plasmid types but still being carried and spread in the same clonal background (SC). Furthermore, horizontal dissemination by larger units than the Tn*1546* as part of a composite transposon between co-existing plasmids has also been previously documented [18, 20]. Moreover, this observation could also explain the mosaicism observed in the plasmidome of hospitalized patients in which plasmid blocks are exchanged between different plasmid types [48].

The dissemination of the *vanA* gene cluster can also occur at a plasmid level in which both plasmid type and Tn*1546* variant are horizontally disseminated, as observed in South-Holland between 2014 and 2015. This type of HGT dissemination can be driven by conjugative plasmids but also from non-conjugative mobilizable plasmids. The presence of non-conjugative plasmids co-residing with conjugative plasmids in the same cell can enhance the horizontal dissemination of both plasmid sequences. This observation has been experimentally validated for the *E. faecium* pHTβ-like plasmid, which contained an efficient conjugation machinery, and allowed the mobilization of other multi-drug resistant and non-conjugative plasmids present in the same cell [49].

The dissemination of vancomycin resistance was previously investigated using WGS in several recent studies [11, 50–53] but seldom with a focus on distinguishing clonal and plasmid outbreaks. One exception is the study by Pinholt et al. [14] that used a combination of short-read and long-read sequencing to describe the clonal expansion of VRE in the Capital Region of Denmark between 2012 and 2015. Here, ST80 was defined as responsible for the first observed local outbreaks. These clonal isolates subsequently spread to other hospitals in the same region. The plasmid bearing the *vanA* gene cluster was disseminated to other, non-clonally related vancomycin-susceptible isolates. In our data set covering the same time frame, ST80 represented by SC18 was also a predominant clone (Fig. 1a).

The emergence of *vanA*-type resistance was also investigated in Australia during 2015 using a combination of short- and long-read sequencing [15]. The study showed the presence of several *vanA* plasmid types which were dominant in each ST group with distinct Tn*1546* variants. This unraveled that, in Australia, the emergence of the *vanA*-type resistance most likely occurred by multiple introductions of different clones which suggested that HGT is not solely responsible for the spread of the *vanA* gene cluster, which is in line with our own results.

Both these studies [14, 15], however, followed a reference-based approach to deduce the presence of a particular *vanA* plasmid type. This could mask the presence of plasmid types which are distinct from the selected reference plasmid(s). In our study, predicted plasmids have been integrated into a network that avoids the arbitrary usage of a reference plasmid and takes plasmid modularity into account. Bipartite networks were previously postulated to explore the pangenome of bacterial species with a particular emphasis on the accessory genome [54]. A network approach also allows classifying plasmids in the absence of common evolutionary history as it can integrate both horizontal and vertical inheritance, in contrast to phylogenetic trees [55, 56]. The classification of plasmids based on k-mer similarity networks has also recently been proposed by Acman et al. [57].

A focus on the core genome can overestimate the number of isolates that are considered as non-related and thus missing potential epidemiological links. In line with Harris et al. [58], we encourage the shift from a traditional core-genome view on outbreak investigations to a new perspective that also includes the analyses of HGT mobilization of AMR genes to effectively confirm potential epidemiological links and correctly evaluate the effectiveness of infection control policies. We showed that highly similar plasmids can be transferred between different SC’s which challenges the interpretation of AMR outbreak studies that are solely focused on core-genome analysis. A factor that contributes to this clonality centricity is the limitations inherent to short-read WGS from which the assembly of plasmids is difficult and error-prone due to the high number of repeated sequences [59]. For full resolution, many studies recommend long-read sequencing to complete chromosomes and plasmids [16, 59, 60]. We show in this study, however, that distinguishing between different modes of spread is feasible also in the absence of long-read data.

A limitation of this work is that only a single colony per isolate was sequenced. This masked the true underlying diversity present in the bacterial population and could have prohibited the detection of potential epidemiological links. While our data set allowed us to define the most likely events of dissemination, we lacked patient admission and ward movement information to discern transmission routes and events, as previously shown by Raven et al. [11] and Neumann et al. [61]. The absence of data on vancomycin-susceptible isolates also prevented us to deduce how the distinct plasmid types and Tn*1546* variants were introduced into the Dutch hospitals. Nonetheless, we succeeded to provide one of the first quantitative assessments to discern the dynamics and contribution of clonal and horizontal transmission in the dissemination of vancomycin resistance.

## Conclusions

This study has shown that in VRE-related cases, clonal dissemination was the preferential mode of dissemination of *vanA*-type vancomycin resistance observed in Dutch hospitals between 2012 and 2015. However, we also detected outbreak settings in which HGT plasmid dissemination contributed most to the spread of resistance. Our analyses showed the importance of taking all nested genomic elements into account to effectively elucidate how resistance spreads in healthcare settings. This is fundamental to corroborate potential epidemiological links that could be neglected by uniquely considering strain relatedness. Only then, the effectiveness of current infection control policies to prevent AMR spread can be truly assessed.

## Supplementary Information

**Additional file 1: Table S1.** Metadata table related to the 309 *vanA*-type vancomycin-resistant *Enterococcus faecium* isolates. (CSV 20 kb)

**Additional file 2.** Supplementary Figures: Fig. S1-Fig. S8.

**Additional file 3. **Supplementary Results: A) Characterization of the sequences defined plasmid types (A-I). B) Characterization of the predicted *vanA* plasmid groups (1–8, > 10 isolates) identified in the network of predicted plasmid bins.

**Additional file 4: Table S2.** Genome assignments and metadata information of the 225 VRE isolates considered to estimate the most likely events of *vanA* dissemination. (CSV 16 kb)

## Data Availability

The raw paired-end reads of the 309 VRE isolates are available through the European Nucleotide Archive project PRJEB28495 [62]. The previous complete *vanA* plasmid sequences (*n* = 26) used to define the plasmid types (A–F) are also available through the European Nucleotide project PRJEB28495 https://www.ebi.ac.uk/ena/browser/view/PRJEB28495 [62] and the gitlab project https://gitlab.com/sirarredondo/vancomycin_dissemination [43]. The plasmid sequences derived from the PLSDB database [34] considered in the manuscript have the following European Nucleotide Archive number accessions: AP022823.1, KX574671.1,KX810025.1,KX810026.1, KX853854.1, KX976485.1, KY554216.1, KY595962.1, KY595966.1, MG674582.1, NC_005054.1, NC_008768.1, NC_008821.1, NC_010980.1, NC_011140.1, NC_013317.1, NC_014475.1, NC_014726.1, NC_014959.1, NC_016967.1, NC_019213.1, NC_019284.1, NC_021170.1, NZ_AP022343.1, NZ_CP011284.1, NZ_CP012431.1, NZ_CP012464.1, NZ_CP012594.1, NZ_CP013996.1, NZ_CP014452.1, NZ_CP014531.1, NZ_CP016166.1, NZ_CP017795.1, NZ_CP018130.1, NZ_CP018825.1, NZ_CP018831.1, NZ_CP019210.1, NZ_CP019973.1, NZ_CP019995.1, NZ_CP020486.1, NZ_CP022486.1, NZ_CP023796.1, NZ_CP025755.1, NZ_CP027500.1, NZ_CP027505.1, NZ_CP027510.1, NZ_CP027516.1, NZ_CP027519.1, NZ_CP033209.1, NZ_CP035644.1, NZ_CP035650.1, NZ_CP036247.1, NZ_CP040238.1, NZ_CP040708.1, NZ_CP041269.1, NZ_CP041279.1 NZ_CP044276.1 NZ_CP046078.1, and NZLR135256.1, NZ_LT603681.1. These PLSDB sequences are also available at https://gitlab.com/sirarredondo/vancomycin_dissemination [43]. The complete code used to generate the results present in this manuscript is provided in a RMarkdown document available through https://gitlab.com/sirarredondo/vancomycin_dissemination [43].

## Abbreviations

WHO: World Health Organization
VRE: Vancomycin-resistant *Enterococcus faecium*
WGS: Whole-genome sequencing
HGT: Horizontal gene transfer
AMR: Antimicrobial resistance
SC: Sequencing cluster
SNP: Single nucleotide polymorphism
ORF: Open reading frame
