# Supplementary material for: Mode and dynamics of vanA-type vancomycin resistance dissemination in Dutch hospitals
Source: Genome Med. 2021 Jan 20;13:9. doi: 10.1186/s13073-020-00825-3 (PMC7816424; doi:10.1186/s13073-020-00825-3)
Supplement: Supplementary file 2 — Additional file 2. Supplementary Figures: Fig. S1-Fig. S8. [file 13073_2020_825_MOESM2_ESM.pdf]

Additional File 2 - Mode and dynamics of *vanA*-type vancomycin resistance dissemination in Dutch hospitals

Authors: Sergio Arredondo-Alonso[1,2], Janetta Top[1], Jukka Corander[2,3,4], Rob J L Willems[1], Anita C Schürch[1]

[1]Department of Medical Microbiology, University Medical Center Utrecht, Utrecht, The Netherlands.

[2]Department of Biostatistics, University of Oslo, Oslo, Norway.

[3]Pathogen Genomics, Wellcome Trust Sanger Institute, Cambridge CB10 1SA, UK.

[4]Department of Mathematics and Statistics, Helsinki Institute of Information Technology (HIIT), FI-00014 University of Helsinki, Finland.

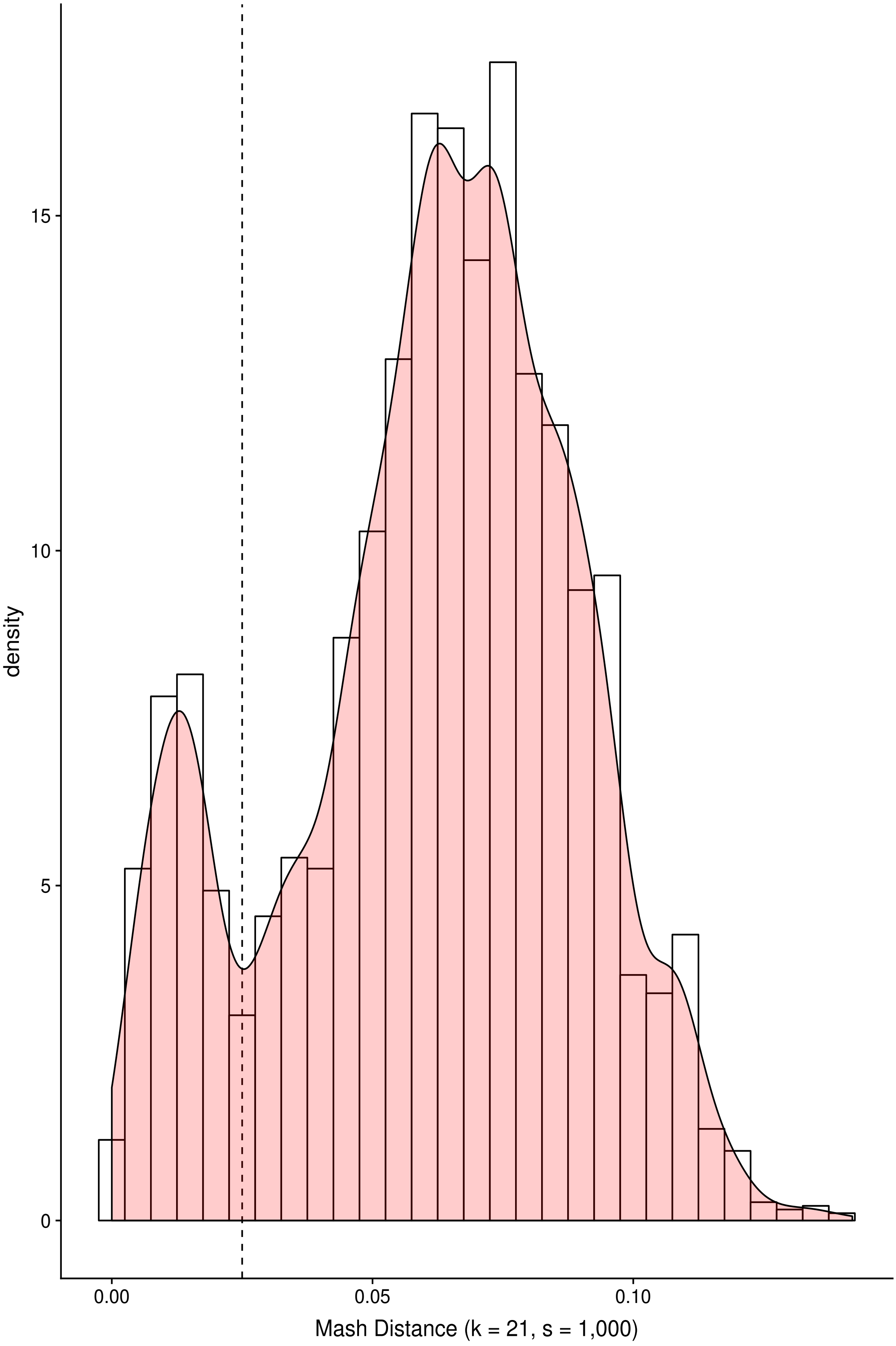

Fig. S1. Distribution of pairwise Mash distances (k = 21, s = 1,000) between *vanA* complete plasmid sequences (n = 86). The vertical dashed line indicates the cutoff (0.025) considered to define an edge in the network.

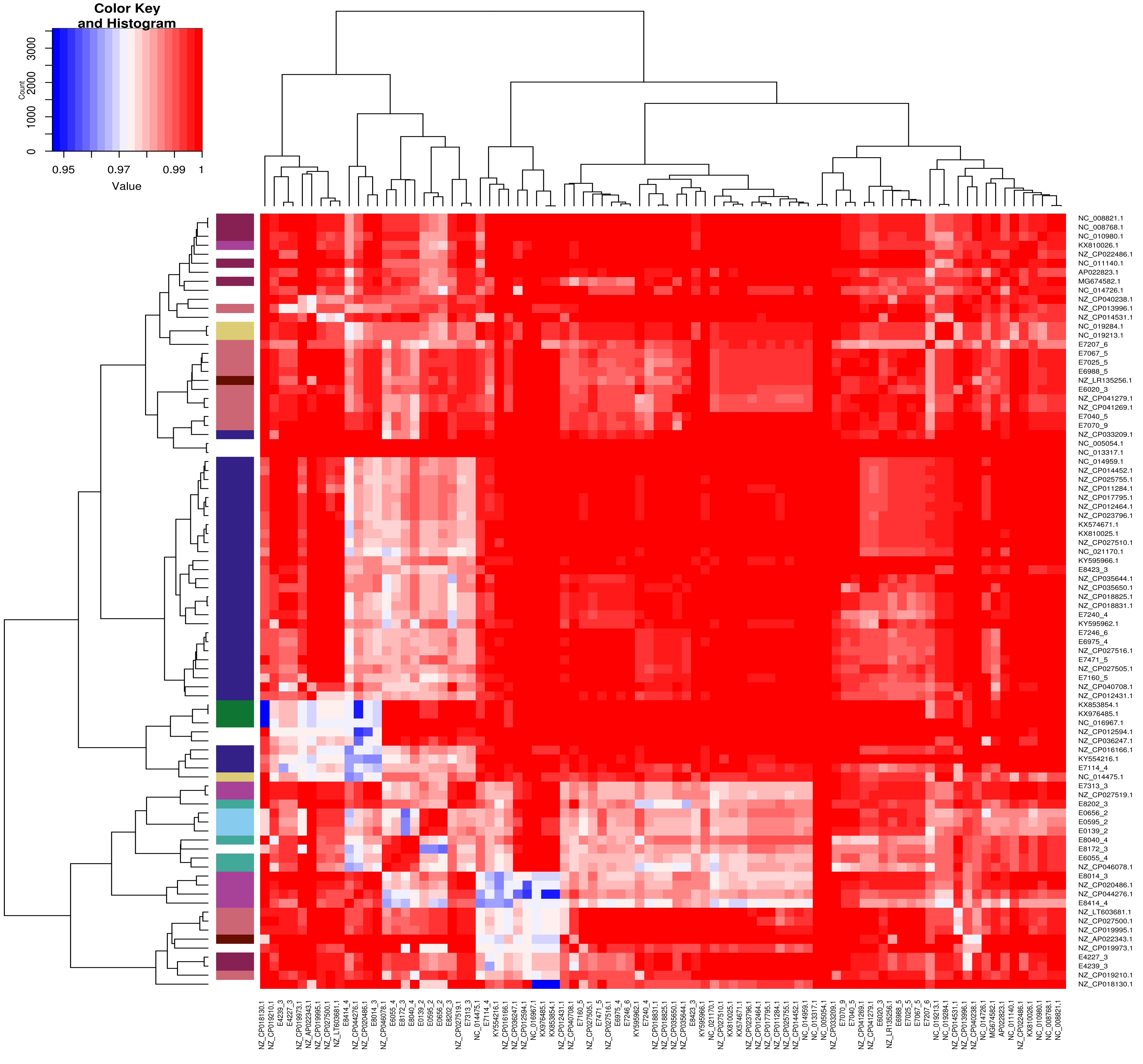

Fig. S2. Heatmap and hierarchical single-linkage clustering (ward.D2 method) of the pyani pairwise alignment identity obtained from the 86 *vanA* complete plasmid sequences. On the left side, the plasmid types (A, B, C, D, E, F, G, H, I) previously defined in the network of Figure 2a are indicated. The twelve complete plasmid sequences corresponding to singletons are also included in this analysis.

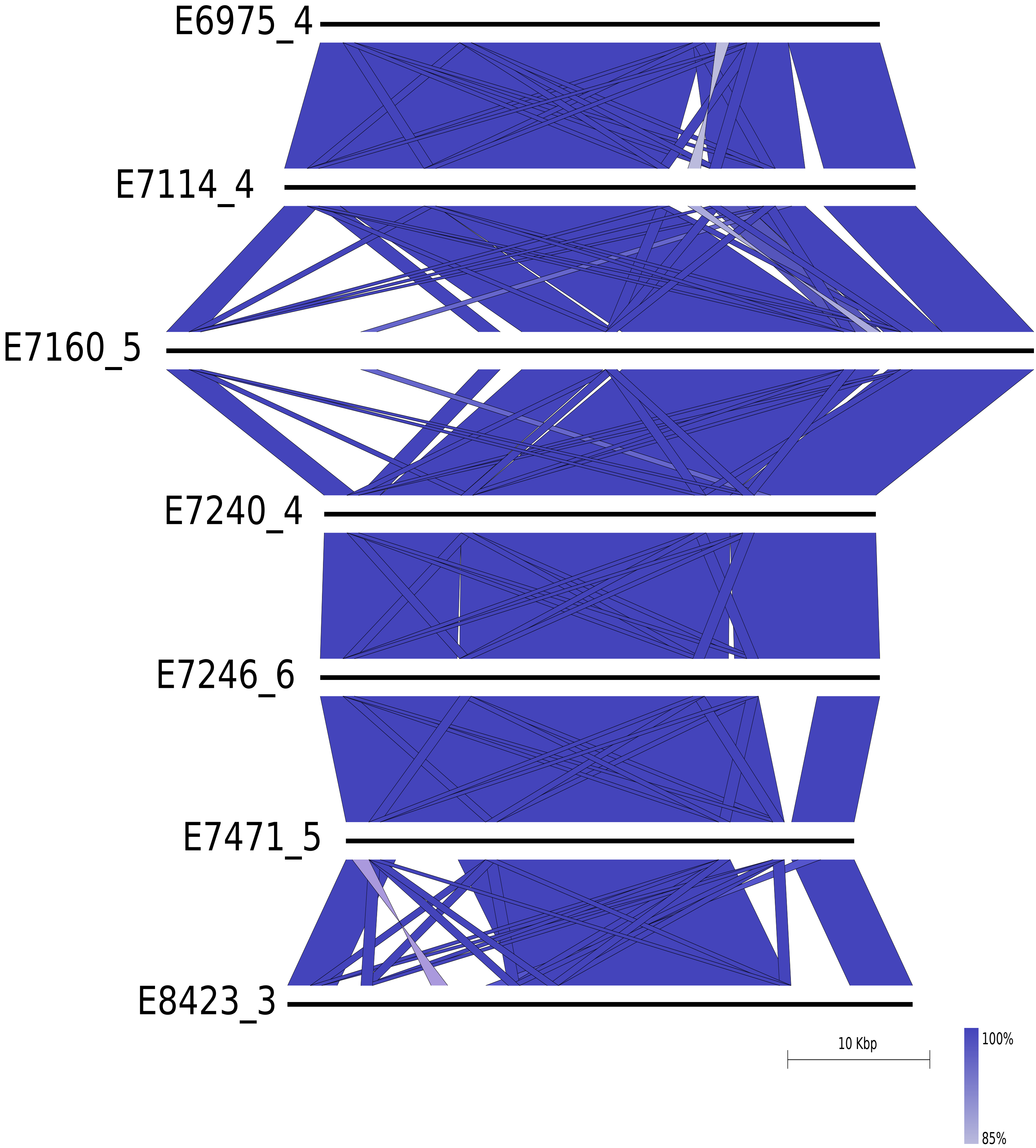

Fig. S3. Visualization of the multiple genome alignment of the complete plasmid sequences described at Arredondo-Alonso et. al 2020 (n = 7) and belonging to the plasmid type B. A minimum blast length of 500 bp and minimum identity of 80% were considered to plot the alignment blocks.

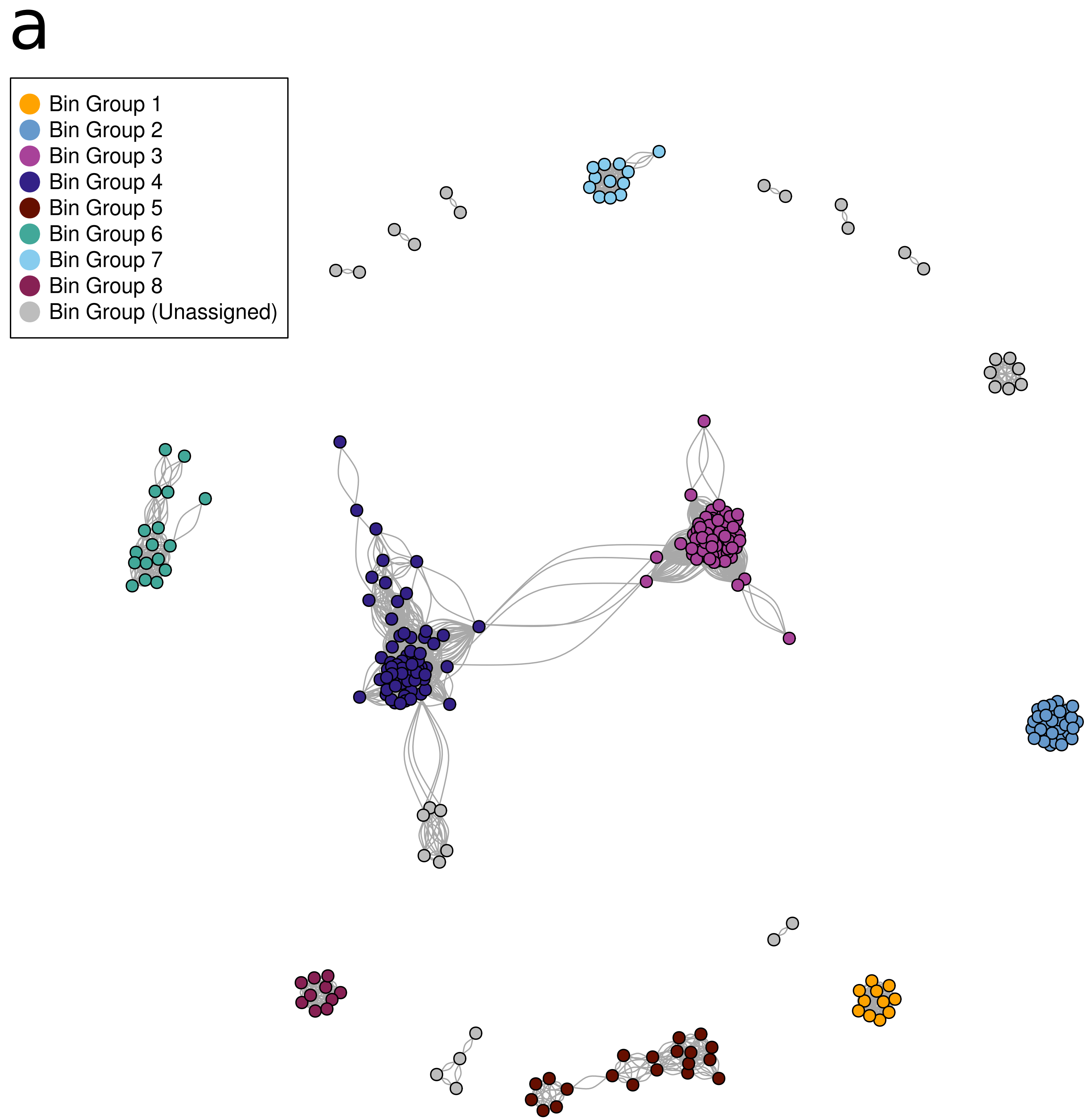

Fig. S4. Network representation of the predicted plasmid bins sequences based on Mash distances (k = 21, s = 1,000). The network consists of 270 nodes corresponding to *vanA* plasmid sequences predicted by gplas. The central component of the network (144 nodes) was split into 3 subgraphs (Louvain method) considering the modularity value of the component. A) Components or subgraphs with more than 10 isolates were assigned as distinct plasmid bin groups (1-8) and coloured accordingly in the network.

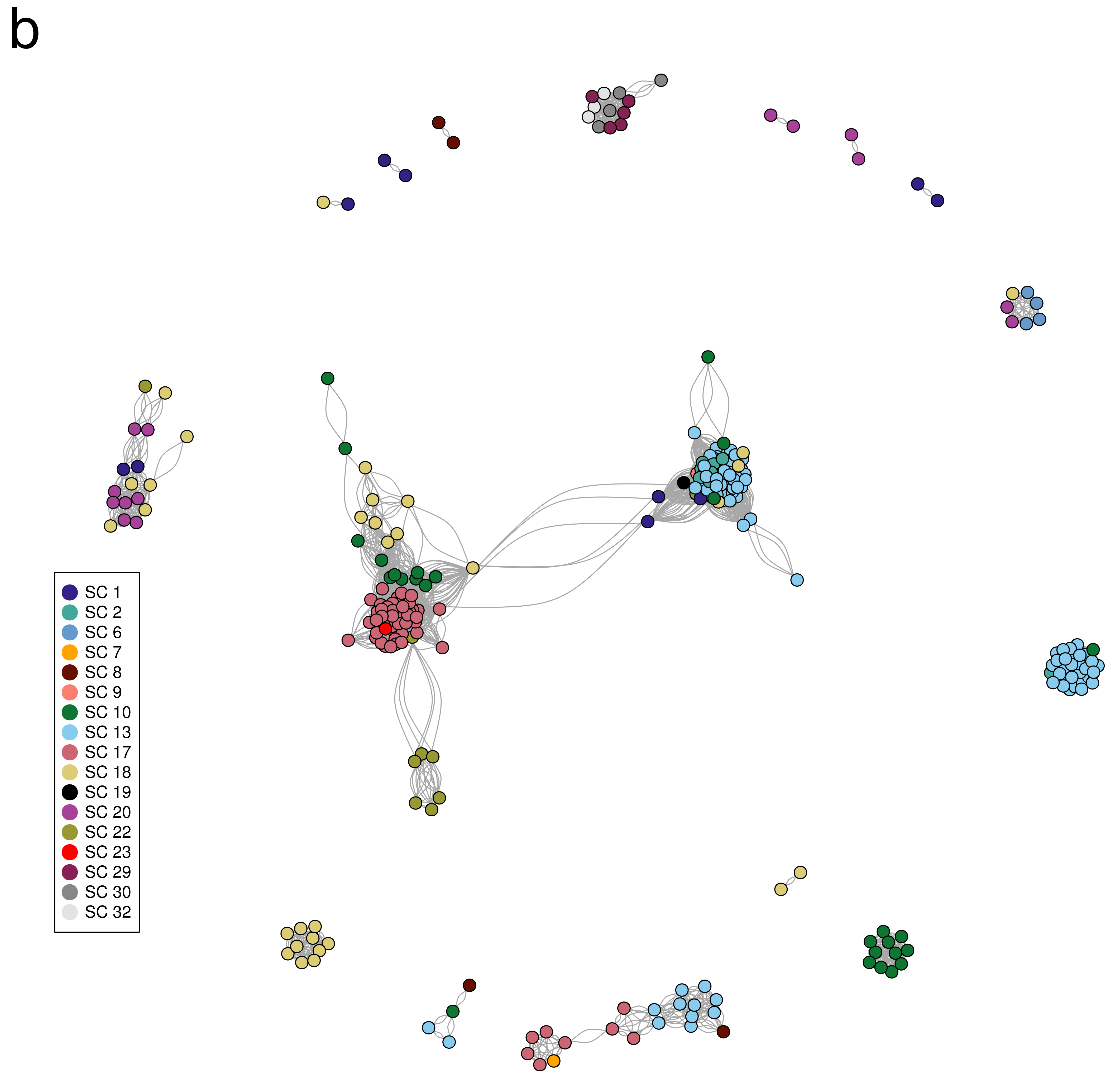

Fig. S4. Network representation of the predicted plasmid bins sequences based on Mash distances (k = 21, s = 1,000). The network consists of 270 nodes corresponding to *vanA* plasmid sequences predicted by gplas. The central component of the network (144 nodes) was split into 3 subgraphs (Louvain method) considering the modularity value of the component. B) Nodes are coloured accordingly to the hierBAPS SC assignment of the isolate carrying the predicted vanA plasmid sequence.

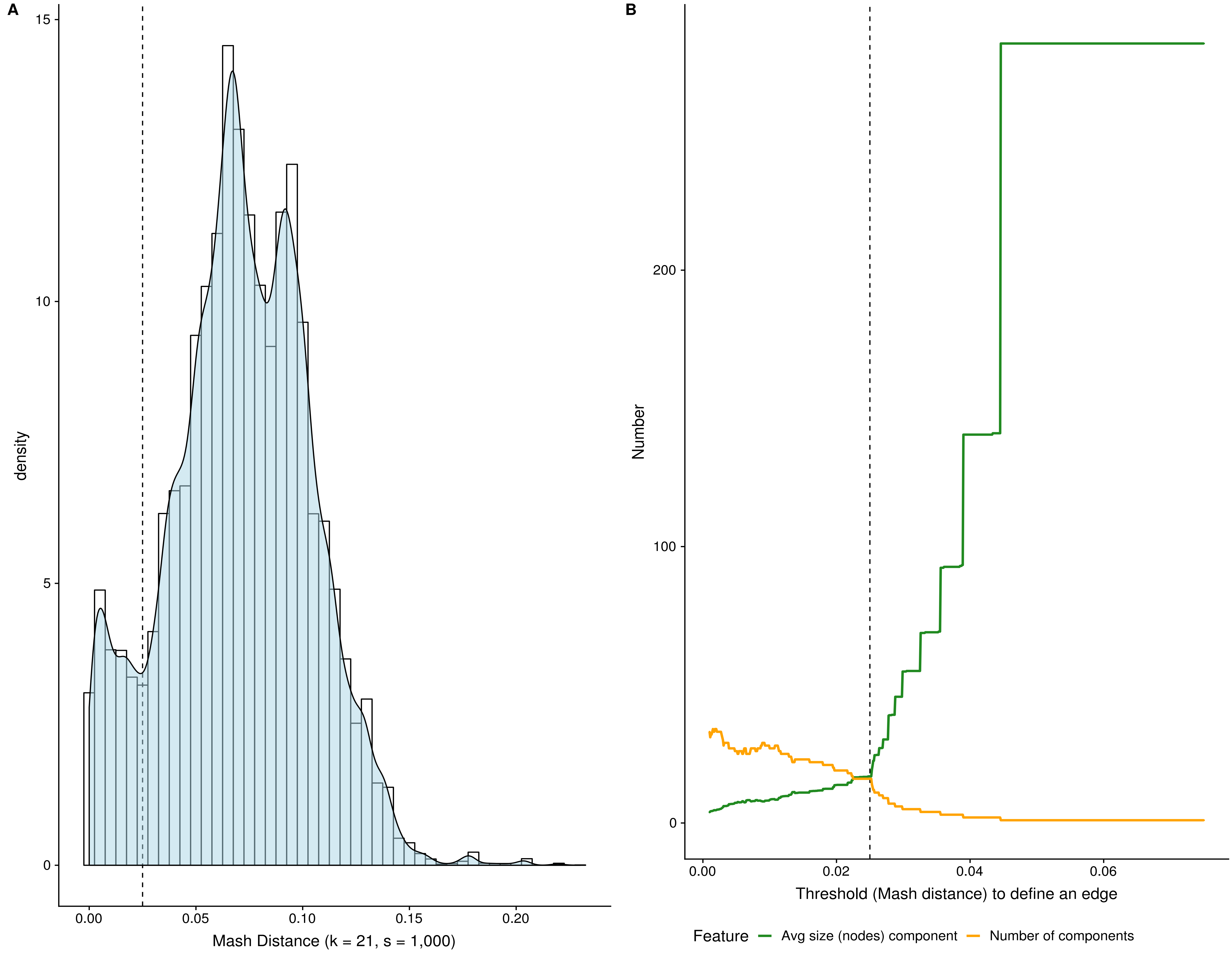

Fig. S5. Distribution of k-mer distances between predicted gplas bins and graph-based analysis performed to define an optimal edge cutoff. A) Distribution of pairwise Mash distances (k = 21, s = 1,000) between predicted *vanA* plasmid sequences (n = 282). A vertical dashed line indicates the cutoff (0.025) considered to define an edge in the network. The distribution resembles the bimodal distribution observed when inspecting the distribution of Mash distances between complete plasmid sequences. B) Average size of the components (green line) and number of components (yellow line) obtained using a continuous range of edge thresholds. The vertical dashed line indicates the cutoff chosen 0.025 which overlaps with the intersection between both lines.

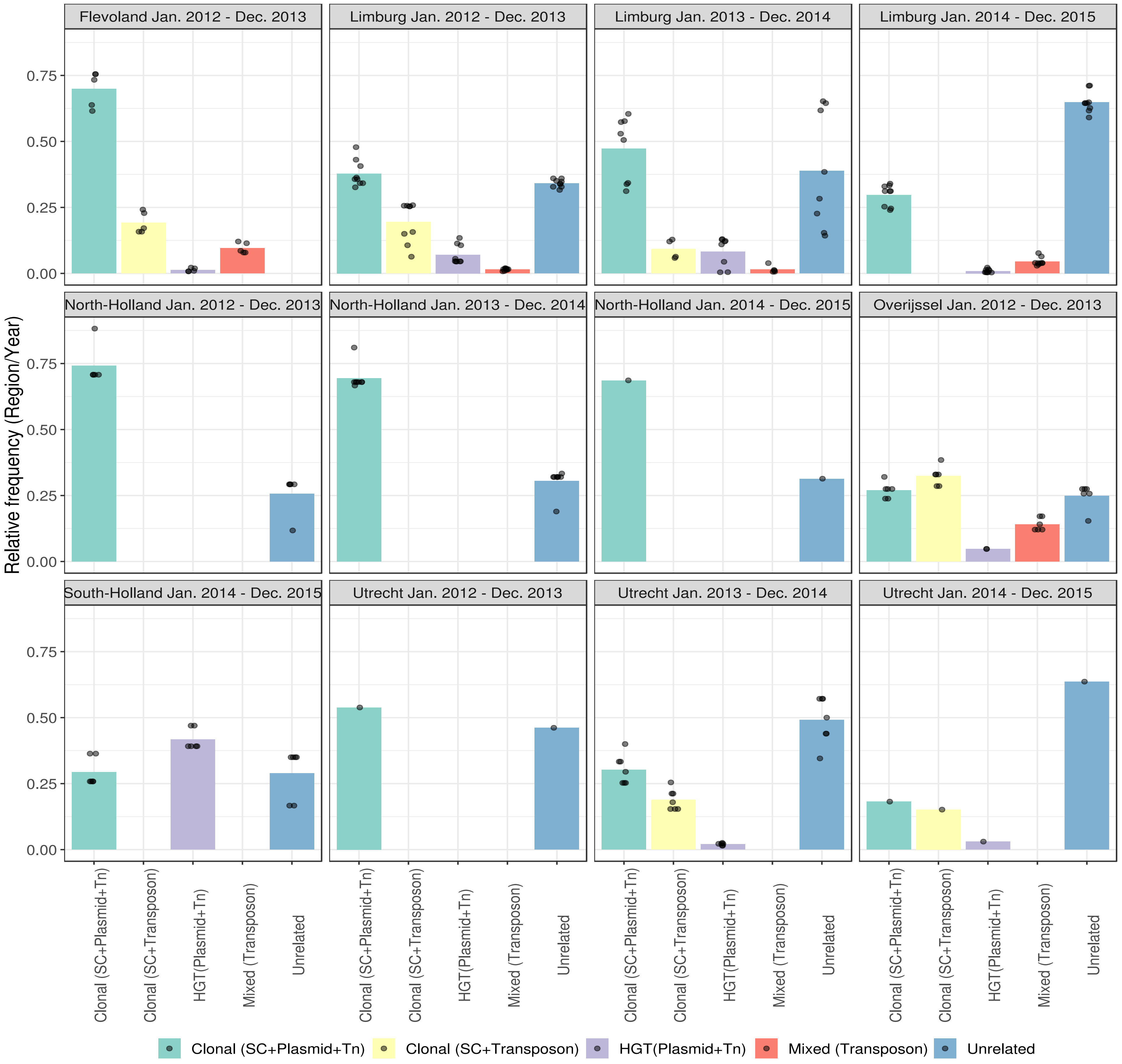

Fig. S6. Dissemination modes in the spread of *vanA*-type vancomycin resistance in the Netherlands between isolates within the same Dutch region. VRE isolates (n = 225) with complete metadata, hierBAPS SC, plasmid type and Tn*1546* assignments were considered. In this analysis, time intervals of two years (Jan. 2012 - Dec. 2013; Jan. 2013 - Dec. 2014; Jan. 2014 - Dec. 2015) were considered to group the isolates. For each time interval, all windows of 12 consecutive months with at least 10 isolates. From each window, we undertook a pairwise comparison between isolates of each window was computed to estimate the frequency of the following events: i) clonal dissemination, pairs of isolates sharing the same hierBAPS SC, *vanA* plasmid type and Tn*1546* variant; ii) plasmid dissemination , pairs of isolates sharing the same *vanA* plasmid type and Tn*1546* variant but distinct hierBAPS SC; iii) clonal dissemination associated with Tn*1546* mobilisation, pairs of isolates sharing the same hierBAPS SC and Tn*1546* variant but different *vanA* plasmid type; iv) Tn*1546* mobilisation linked to clonal and horizontal dissemination, pairs of isolates sharing the same Tn*1546* variant and distinct hierBAPS SC and *vanA* plasmid type; and v) unrelated cases, pairs of isolates with distinct Tn*1546* variants.

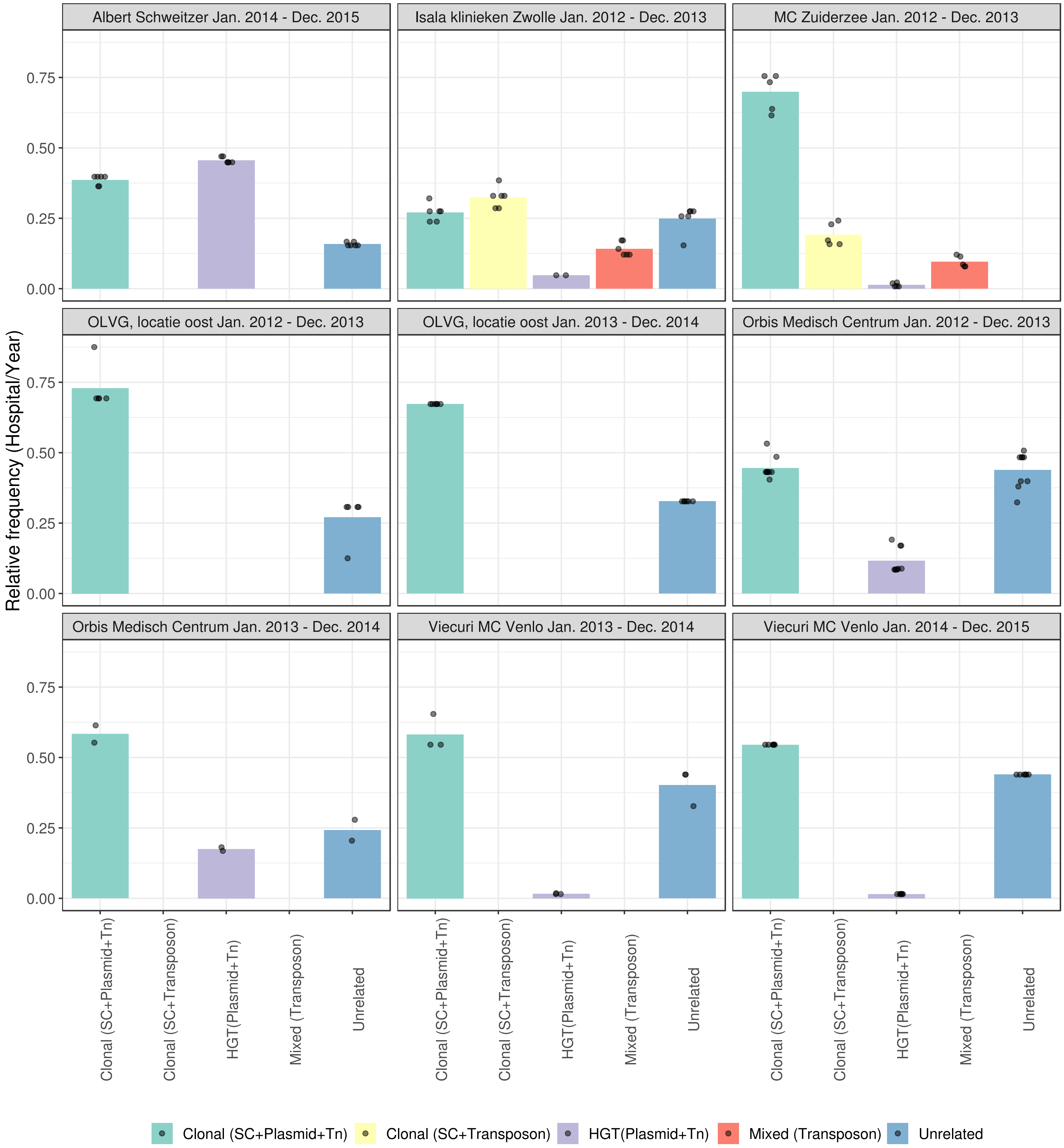

Fig. S7. Dissemination modes in the spread of *vanA*-type vancomycin resistance in the Netherlands between isolates within the same hospital. VRE isolates (n = 225) with complete metadata, hierBAPS SC, plasmid type and Tn*1546* assignments were considered. In this analysis, time intervals of two years (Jan. 2012 - Dec. 2013; Jan. 2013 - Dec. 2014; Jan. 2014 - Dec. 2015) were considered to group the isolates. For each time interval, all windows of 12 consecutive months with at least 10 isolates. From each window, we undertook a pairwise comparison between isolates of each window was computed to estimate the frequency of the following events: i) clonal dissemination, pairs of isolates sharing the same hierBAPS SC, *vanA* plasmid type and Tn*1546* variant; ii) plasmid dissemination , pairs of isolates sharing the same *vanA* plasmid type and Tn*1546* variant but distinct hierBAPS SC; iii) clonal dissemination associated with Tn*1546* mobilisation, pairs of isolates sharing the same hierBAPS SC and Tn*1546* variant but different *vanA* plasmid type; iv) Tn*1546* mobilisation linked to clonal and horizontal dissemination, pairs of isolates sharing the same Tn*1546* variant and distinct hierBAPS SC and *vanA* plasmid type; and v) unrelated cases, pairs of isolates with distinct Tn*1546* variants.

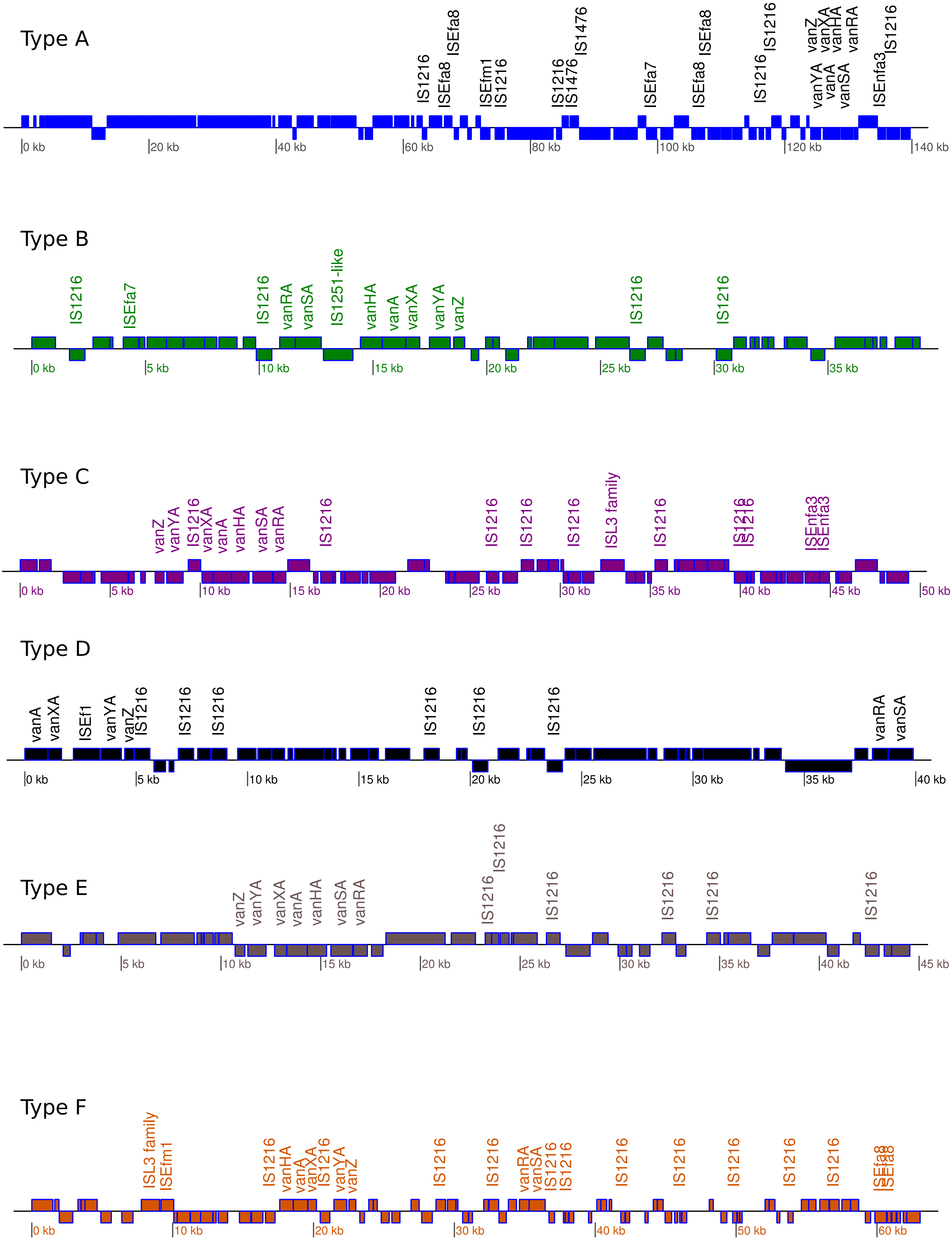

Fig. S8. Scheme of the gene structure and content present in the plasmid types (A-F) inferred from plasmid sequences described at Arredondo-Alonso et. al 2020. For visualization purposes, only the genes belonging to the *vanA* cluster and IS elements present in the plasmids are indicated.
