# Supplementary material for: Mode and dynamics of vanA-type vancomycin resistance dissemination in Dutch hospitals
Source: Genome Med. 2021 Jan 20;13:9. doi: 10.1186/s13073-020-00825-3 (PMC7816424; doi:10.1186/s13073-020-00825-3)
Supplement: Supplementary file 3 — Additional file 3. Supplementary Results: A) Characterization of the sequences defined plasmid types (A-I). B) Characterization of the predicted vanA plasmid groups (1–8, > 10 isolates) identified in the network of predicted plasmid bins. [file 13073_2020_825_MOESM3_ESM.pdf]

Additional File 3 : Supplementary Results - Mode and dynamics of *vanA*-type vancomycin resistance dissemination in Dutch hospitals

Authors: Sergio Arredondo-Alonso[1,2], Janetta Top[1], Jukka Corander[2,3,4], Rob J L Willems[1], Anita C Schürch[1]

[1]Department of Medical Microbiology, University Medical Center Utrecht, Utrecht, The Netherlands.
[2]Department of Biostatistics, University of Oslo, Oslo, Norway.
[3]Pathogen Genomics, Wellcome Trust Sanger Institute, Cambridge CB10 1SA, UK.
[4]Department of Mathematics and Statistics, Helsinki Institute of Information Technology (HIIT), FI-00014 University of Helsinki, Finland.

A) Characterization of the genomic organization of the plasmid types

The network approach, based on complete plasmid sequences from Arredondo-Alonso et. al 2020 [1] (n =26) and PLSDB plasmid sequences (n = 60), allowed the identification of nine distinct plasmid types (A-I). Next, we describe the content of the complete plasmid sequences from Arredondo-Alonso et. al 2020 [1] in the plasmid types (A-I). These sequences were selected to highlight the plasmid content since we had available: i) replication initiator protein information, ii) AMR gene content information and iii) metadata information regarding isolation source and isolation year. In addition, for these isolates we had available short-read sequencing data which was required to run TETyper [2] and compute the Tn*1546* variant present in these plasmids. We explore the plasmid content with a focus on: i) replication initiator proteins (rep), ii) Tn*1546* variant compared to the original sequence described by Arthur et al. [3], iii) AMR genes distinct from the *vanA* gene cluster and iv) presence of well-known *E. faecium* plasmid TA systems such as ω-ε-ζ and axe-txe [4].

The plasmid type A was highly similar to large RepA plasmids with a size ranging from 141.9 kbp to 189.4 kbp (mean = 157.1 kbp, median = 141.9 kbp). A similar-sized RepA plasmid was also previously identified, using pulsed-field gel electrophoresis patterns, in pig and healthy

human isolates recovered in the same time period from Denmark, Portugal, Spain, Switzerland, and the United States [5]. The plasmid described in the previous study [5] carried Tn*1546* variant, termed 'D' [5,6], which corresponded to deletions in *orf1* (1-199) and the SNP position G8234T. In our study, type A plasmids showed the same Tn*1546* variant. However, the Tn*1546* variant present in plasmid E0656_2 carried an extra SNP position C4947T . Tn*1546* is flanked by IS1216 elements upstream and downstream from the transposon (Additional File 2 : Fig. S8). This RepA megaplasmid also encodes for the *erm(B)* gene conferring resistance to erythromycin and a copper resistance operon *tcrYAZB* which has been shown to be implicated in tolerance to high levels of this heavy metal [1]. This plasmid also encodes the TA system ω-ε-ζ previously described in poultry isolates [7]. The mobilisation of this plasmid type between distinct hosts, healthy humans and pigs, could be explained by the presence of the type IV secretion gene *traG*. This *traG* gene has been previously reported in pIP501, a conjugative broad-host-range plasmid present in nosocomial *E. faecium* and *E. faecalis* isolates [8].

The plasmid type B was composed of 31 sequences which included 24 PLSDB complete plasmid sequences (KX574671.1, KX810025.1, KY554216.1, KY595962.1, KY595966.1, NC_014959.1, NC_021170.1, NZ_CP011284.1, NZ_CP012431.1, NZ_CP012464.1, NZ_CP014452.1, NZ_CP016166.1, NZ_CP017795.1, NZ_CP018825.1, NZ_CP018831.1, NZ_CP023796.1, NZ_CP025755.1, NZ_CP027505.1, NZ_CP027510.1, NZ_CP027516.1, NZ_CP033209.1, NZ_CP035644.1, NZ_CP035650.1, NZ_CP040708.1) and seven plasmid sequences previously described in Arredondo-Alonso et. al 2020 (E6975_4, E7114_4, E7160_5, E7240_4, E7246_6, E7471_5, E8423_3). In these seven plasmid sequences, we found that plasmid type B is a multireplicon RepA_N & Rep_3-like plasmid with a length ranging from 35.7 kbp to 61.1 kbp. The Tn*1546* variant present in this type is characterized by the insertion of the IS*1251*-like element (5820) and SNPs at positions T7658C, G8234T and C9692T. Two plasmids, E7246_6 and E8423_3, showed the additional SNP G4351T. We also observed deletions in *orf1*,*orf2* mostly between the coordinates 1-3702 but also in the coordinates 1-3343. This Tn*1546* variant was previously termed as 'C' because of the presence of the IS*1251*-like element [9]. Also in this plasmid, *Tn1546* was surrounded by two IS*1216* elements (Additional File 2 : Fig. S8). Plasmid type B also contained other AMR genes including: i) *erm(B)*, erythromycin resistance, ii) *aph(3')*, aminoglycoside resistance and iii) *cfr(C)*, linezolid resistance. In addition, this plasmid contains the TA axe-txe complex first described in the *E. faecium* pRUM plasmid [10].

The plasmid type C was composed of seven sequences including four PLSDB complete plasmid sequences (KX810026.1, NZ_CP020486.1, NZ_CP027519.1, NZ_CP044276.1) and three plasmid

sequences described in Arredondo-Alonso et. al 2020 (E7313_3, E8014_3, E8414_4). Based on these three last sequences, we could define plasmid type C as a multireplicon Inc18 & Rep_trans plasmids ranging in size from 42.6 kbp to 49.9 kbp (mean = 45.1 kbp, median = 42.7 kbp). The Tn*1546* variant observed in this plasmid contained two IS*1216* elements inserted in positions 3932 and 8646, respectively. In addition, we observed deletions in *orf1, orf2* (1-3676) and in the intergenic region 8650-8827 or 8650-8925. As previously described, the specific coordinates of the deletions could differ between the isolates while the rest of SNP coordinates or IS-elements were identical. In this plasmid type, we also observed the presence of other AMR genes including *ant(6)-Ia* and *erm(B)* conferring resistance to aminoglycoside and erythromycin, respectively.

Plasmid type D is an Inc18 plasmids with a size ranging from 40.2 kbp to 47.9 kbp (mean = 43.1.5 kbp, median = 41.1 kbp). The Tn*1546* variant present in this plasmid type contained an insertion of ISEf1 (9051) for the variants present in the plasmid E6055_4 and E8040_4 whereas the SNP G7747T was found in all variants. However, the coordinates of deletions present in Tn*1546* were different between the isolates bearing the plasmid type D. The Tn*1546* variant present in the plasmid E6055_4 showed a deletion in *vanZ* (10490-10850), the transposon variant in the plasmid E8040_4 presented deletions in *vanS* (5895-5932) and the intergenic region (10706-10850) whereas the variant found in E8202_3 presented a deletion between the genomic coordinates 9027-10850 . In the plasmid type D, the Tn*1546* variant showed the presence of an IS*1216* element downstream of the *vanZ* gene (Additional File 2 : Fig. S8). The plasmid type D also encoded the *erm(B)* gene conferring resistance to erythromycin.

Plasmid type E included seven plasmid sequences, five from the PLSDB database (MG674582.1 , NC_008768.1, NC_008821.1, NC_010980.1, NC_011140.1) and two previously described at Arredondo-Alonso et. al 2020 (E4227_3, E4239_3). In these 2 plasmid sequences, we observed that the plasmid type E was a multireplicon Inc18 & Rep3 plasmid with a size ranging from 45 kbp to 46 kbp (mean = 45.8 kbp, median = 45.8 kbp). We observed the original Tn*1546* variant, also termed as 'A', with no deletions, IS elements or point mutations present in comparison to the sequence described by Arthur et al. [3]. This plasmid has been previously described as pVEF4 and characterized in poultry isolates from Norway and Denmark [11]. Plasmid type E reported here, contained functional and truncated *str* genes conferring resistance to streptomycin. We uniquely observed two components, ω- ε , of the TA system previously described in *E. faecium* plasmid sequences derived from poultry isolates [7].

Plasmid type F was composed of 14 plasmid sequences including seven PLSDB complete plasmid sequences (NZ_CP013996.1, NZ_CP019210.1, NZ_CP019995.1, NZ_CP027500.1, NZ_CP041269.1, NZ_CP041279.1, NZ_LT603681.1) and seven plasmid sequences described in Arredondo-Alonso et. al 2020 (E6020_3, E6988_5, E7020_5, E7040_5, E7067_5, E7070_9, E7207_6). In these last 7 plasmid sequences, we could characterize the plasmid type F as a multireplicon Inc18 & Rep3-like plasmid, with an additional Rep2-like element in some isolates. The size of the plasmids ranged from 38.6 kbp to 63.68 kbp (mean = 48.2 kbp, median = 48.2 kbp). This plasmid contained a Tn*1546* variant carrying IS*1216* insertions at positions 5768 and 8839 with a deletion in *orf1* (1-2547). The IS*1216* insertion in the position 8839 has been previously reported [12]. Notably, in the plasmid type F, the Tn*1546* element was split into blocks which were surrounded by IS*1216* elements (Additional File 2 : Fig. S8). The *vanR* and *vanS* genes were separated (> 10 kbp) from the rest of the *vanA* gene cluster (Additional File 2 : Fig. S8). In some plasmids E6020_3, E6988_5, E7025_5 and E7067_5, we observed other AMR genes including *erm(B)*, *aph(3')-III*, *ant(6)-Ia* and *lnu(B)* linked to the co-integration of a Rep2-like plasmid. In all the plasmids from this type, we observed the presence of the TA system ω-ε-ζ .

Lastly, we observed three plasmid types (G, H, I) only composed of PLSDB plasmid sequences. Plasmid type G formed by three PLSDB sequences: KX853854.1, KX976485.1, NC_016967.1. Plasmid type H which included three PLSDB sequences: NC_014475.1 NC_019213.1, NC_019284.1. Lastly, plasmid type I formed by two PLSDB sequences: NZ_AP022343.1, NZ_LR135256.1

B) Characterization of the predicted plasmid bins

To elucidate gene content and synteny of these plasmid bins, we integrated the nine plasmid types (A-I) derived from complete plasmid sequences (Table 1, Fig. 2) with the plasmid bins predicted by gplas (Additional File 2 : Fig. S3). We calculated Mash distances (k = 21, s = 1,000) between the complete *vanA* plasmid sequences (n = 26), PLSDB retrieved plasmid sequences (n = 60), and the predicted *vanA* plasmid bins (n = 282). The presence of edges connecting complete plasmids and predicted *vanA* plasmid bins revealed that the predictions had a similar k-mer content and thus further validated the predicted *vanA* plasmid bins (Additional File 2 : Fig. S3). Furthermore, this approach also allowed to elucidate the content and structure of the plasmids present in the predicted network. Due to the fragmented nature of the predicted plasmid bins, the Tn*1546* characterisation was performed considering uniquely SNP and deletions respect to the original transposon sequence described by Arthur et al [3].

We focused on the presence of eight distinct groups of plasmid bins which corresponded to subgraphs highly interconnected in the network with more than 10 nodes (Additional File 2 : Fig. S3a, Table 2).

Predicted group (plasmid bins) 1 represented a novel plasmid type, termed J, which was not linked to one of the nine plasmid-types included (A-I) (Fig. 3). This was the only predicted group without a complete sequence co-clustering in the network. Isolates carrying this novel plasmid type J (n = 11) belonged to SC10 (Table 2). The majority of the isolates were from Utrecht and isolated in April-May 2012 (n = 9). However, we also found two isolates from May 2013 (E7837) and March 2014 (E8046) which were present in nearby Dutch cities (Ede - Gelderland region and Amersfoort - Utrecht region). All isolates (n = 11, 100%) shared the same Tn*1546* variant (MN) with the SNPs T7658C and G8234T.

Plasmid bins in the group 3 co-clustered with plasmid type C (n = 76) (Fig. 3). Isolates carrying this plasmid type belonged mostly to four distinct SCs: 13,12,1,10 (Table 2) which suggested horizontal spread of the plasmid type C between non-clonal isolates. Furthermore, the predicted plasmids were widely observed in the Netherlands during the entire collection period (2012-2015). Tn*1546* characterisation identified the variant MNI characterised by deletions in the *orf1*, *orf2* region (1-3417 in 53.1% and 1-3676 in 32.8% cases) and in the intergenic region (8650-8827). We observed an independent plasmid bin group 2 mainly formed by isolates belonging to SC13 (Table 2) but geographical widespread in the Netherlands between 2012 and 2015 (Fig. 1). Detailed analysis of the predicted plasmid types of isolates E7313, E8014, E8414 with associated complete plasmid sequences, revealed that these plasmids were linked to both plasmid bin groups 2 and 3, while their complete plasmid sequences belonged to a single plasmid type C. Furthermore, both predicted *vanA* plasmid bins (2, 3) shared the same Tn*1546* variants. These observations suggested that the predicted plasmid bins groups 2 and 3 should be merged and named type-C.

Plasmid bins in the group 4 co-clustered with plasmid type B (n = 62) (Fig. 3). Isolates carrying this plasmid type belonged to three different SCs (10,17,18) and were isolated from different Dutch cities (Amsterdam, Lelystad, Zwolle, Utrecht, Nieuwegein and Rotterdam) in 2012. Most of the predicted plasmids (n = 45, 72.6%) exhibited the SNP positions T7658C and G8234T (46 variant) and six isolates (9.7%) had the additional SNP position C9692T (468 variant). These SNP positions coincide with the Tn*1546* variant previously reported for plasmid type B. In

addition, deletions in *orf1* and *orf2*, with coordinates 1-3343, were identified in 88.2% of the cases.

Plasmid bins belonging to the group 5 shared connections to PLSDB sequences defining the plasmid type I (NZ_AP022343.1, NZ_LR135256.1) (Fig. 3). In this group, the isolates belonged to two predominant SCs, SC13 and SC17. (Table 2). A close inspection of the Tn*1546* variants revealed two variants MNI (n = 10, 52.6%) and 46MN (n = 9, 47.4%). The predicted plasmid type 5 was mainly represented by isolates from 2012 (80%) and was present in distinct Dutch provinces (Limburg, Utrecht, Flevoland and Overijssel).

Plasmid bins belonging to the group 6 co-clustered with plasmid type D (Fig. 3). Isolates carrying this plasmid type mainly belonged to isolates with SC20, SC18, SC1, and SC22. They were located widespread around the Netherlands, and retrieved between 2012-2015. The most predominant Tn*1546* variant present in this plasmid type (82.4% cases) contained the G7747T SNP and deletions in the intergenic regions 5896-5931 and 10706-10851 (5II variant).
Plasmid bins in the group 7 co-clustered in the network together with plasmids type A (n = 12) (Fig. 3). Isolates carrying this plasmid type belonged to three distinct SCs : SC29, SC30, and SC32 (Table 2). All isolates were recovered from a single Dutch province (North Holland) spanning three years (2012 to 2015). The Tn*1546* characterisation revealed that all plasmids (predicted and completed) shared a SNP position G8234T and deletion of orf1 (1-119) (6M variant).

The predicted group 8 shared connections with the plasmid type termed E. However, we only observed single node in the component connected to the complete sequences co-clustering with plasmid type E (NC_008768.1, NC_008821.1, NC_011140.1) which indicates that this predicted group 8 may share some k-mer modules with the plasmid type E. All isolates (n = 10) carrying this *vanA* plasmid type belonged to SC18 and were isolated in Venlo (Limburg province) during September-October 2014. Tn*1546* identified in this plasmid was identical to the original transposon described by Arthur et al. [3].

Plasmid types G and H, identified in the fully assembled strains, did not have connections to the predicted plasmid bins in the network (Fig. 3). This suggested that these plasmid types were clearly distinct to the predicted plasmid bins and were not present in the Dutch collection.

Supplementary References

1. Arredondo-Alonso S, Top J, McNally A, Puranen S, Pesonen M, Pensar J, et al. Plasmids Shaped the Recent Emergence of the Major Nosocomial Pathogen *Enterococcus faecium*. MBio [Internet]. 2020;11. Available from: http://dx.doi.org/10.1128/mBio.03284-19

2. Sheppard AE, Stoesser N, German-Mesner I, Vegesana K, Sarah Walker A, Crook DW, et al. TETyper: a bioinformatic pipeline for classifying variation and genetic contexts of transposable elements from short-read whole-genome sequencing data. Microb Genom [Internet]. 2018;4. Available from: http://dx.doi.org/10.1099/mgen.0.000232

3. Arthur M, Molinas C, Depardieu F. Characterization of Tn1546, a Tn3-related transposon conferring glycopeptide resistance by synthesis of depsipeptide peptidoglycan precursors in *Enterococcus faecium* BM4147. Journal of Bacteriology. Am Soc Microbiol; 1993;175:117–27.

4. Fernández-García L, Blasco L, Lopez M, Bou G, García-Contreras R, Wood T, et al. Toxin-Antitoxin Systems in Clinical Pathogens. Toxins [Internet]. 2016;8. Available from: http://dx.doi.org/10.3390/toxins8070227

5. Freitas AR, Coque TM, Novais C, Hammerum AM, Lester CH, Zervos MJ, et al. Human and swine hosts share vancomycin-resistant *Enterococcus faecium* CC17 and CC5 and *Enterococcus faecalis* CC2 clonal clusters harboring Tn*1546* on indistinguishable plasmids. J Clin Microbiol. 2011;49:925–31.

6. Jensen LB, Poulsen RL, Westh H, Hammerum AM. Vancomycin-Resistant *Enterococcus faecium* Strains with Highly Similar Pulsed-Field Gel Electrophoresis Patterns Containing Similar Tn*1546*-Like Elements Isolated from a Hospitalized Patient and Pigs in Denmark. Antimicrobial Agents and Chemotherapy. 1999;43:724–5.

7. Sletvold H, Johnsen PJ, Hamre I, Simonsen GS, Sundsfjord A, Nielsen KM. Complete sequence of *Enterococcus faecium* pVEF3 and the detection of an omega-epsilon-zeta toxin-antitoxin module and an ABC transporter. Plasmid. 2008;60:75–85.

8. Arends K, Celik E-K, Probst I, Goessweiner-Mohr N, Fercher C, Grumet L, et al. TraG encoded by the pIP501 type IV secretion system is a two-domain peptidoglycan-degrading enzyme essential for conjugative transfer. J Bacteriol. 2013;195:4436–44.

9. Wardal E, Kuch A, Gawryszewska I, Żabicka D, Hryniewicz W, Sadowy E. Diversity of plasmids and Tn*1546*-type transposons among *VanA Enterococcus faecium* in Poland. Eur J Clin Microbiol Infect Dis. 2017;36:313–28.

10. Grady R, Hayes F. Axe-Txe, a broad-spectrum proteic toxin-antitoxin system specified by a multidrug-resistant, clinical isolate of *Enterococcus faecium*. Mol Microbiol. 2003;47:1419–32.

11. Leinweber H, Alotaibi SMI, Overballe-Petersen S, Hansen F, Hasman H, Bortolaia V, et al. Vancomycin resistance in *Enterococcus faecium* isolated from Danish chicken meat is located on

a pVEF4-like plasmid persisting in poultry for 18 years. Int J Antimicrob Agents. 2018;52:283–6.

12. Novais C, Freitas AR, Sousa JC, Baquero F, Coque TM, Peixe LV. Diversity of Tn*1546* and its role in the dissemination of vancomycin-resistant enterococci in Portugal. Antimicrob Agents Chemother. 2008;52:1001–8.
